# Laccase-mediated chemoselective C-4 arylation of 5-aminopyrazoles

**DOI:** 10.1371/journal.pone.0308036

**Published:** 2024-09-18

**Authors:** Mansour Shahedi, Rojina Shahani, Niloofar Omidi, Zohreh Habibi, Maryam Yousefi, Mehdi Mohammadi

**Affiliations:** 1 Department of Organic Chemistry, Shahid Beheshti University, Tehran, Iran; 2 Nanobiotechnology Research Center, Avicenna Research Institute, ACECR, Tehran, Iran; 3 Bioprocess Engineering Department, Institute of Industrial and Environmental Biotechnology, National Institute of Genetic Engineering and Biotechnology (NIGEB), Tehran, Iran; Vignan Pharmacy College, INDIA

## Abstract

Chemoselective arylation of 5-aminopyrazoles was performed through oxidative formation of orthoquinones from catechols catalyzed by *Myceliophthora thermophila* laccase (Novozym 51003), and subsequently nucleophilic attack of 5-aminopyrazole to the catechol intermediates. The C-4 arylated products were obtained under extremely mild conditions without the need for amine protection or halogenation of the substrates. From this method, 10 derivatives with moderate to good efficiency (42–94%) were prepared.

## Introduction

The synthesis of heterocyclic compounds or their derivatives is of great interest in organic chemistry due to their diverse properties [1, 2]. Heterocycles that contain nitrogen are very important due to their presence in various medicinal and natural compounds, and finding new routes for expanding the library of these compounds has been the subject of many studies [3, 4]. Pyrazoles are five-membered aromatic heterocyclic rings made up of three carbon atoms and two neighboring nitrogen atoms [5–8]. 5-Amino pyrazoles are a subclass of pyrazoles that contain an amino group (-NH_2_) linked to the pyrazole ring [9, 10]. 5-Amino pyrazoles have shown ant-inflammatory [11], anti-cancer [12] and anti-microbial [13, 14] properties.

5-Aminopyrazoles functionalization is of great interest in pharmaceutical and material chemistry, where the goal is to develop novel molecules with improved properties [15–19]. Acylation [20], alkylation [21–23, sulfonylation [24], cyclization [25, 26], and arylation [27, 28] are some popular techniques for functionalization of 5-aminopyrazoles. These reactions can produce amides, alkylated amines, sulfonyl amines, pyrazolo[1,5-a] pyrimidines, and arylated pyrazoles, respectively. Recently, the C-4 functionalized 5-aminopyrazoles have been proved to present anti-inflammatory properties [29], inhibitory effect on cyclin-dependent kinases [30], and anti-proliferative activity against MCF7 [31].

**Fig 1:**
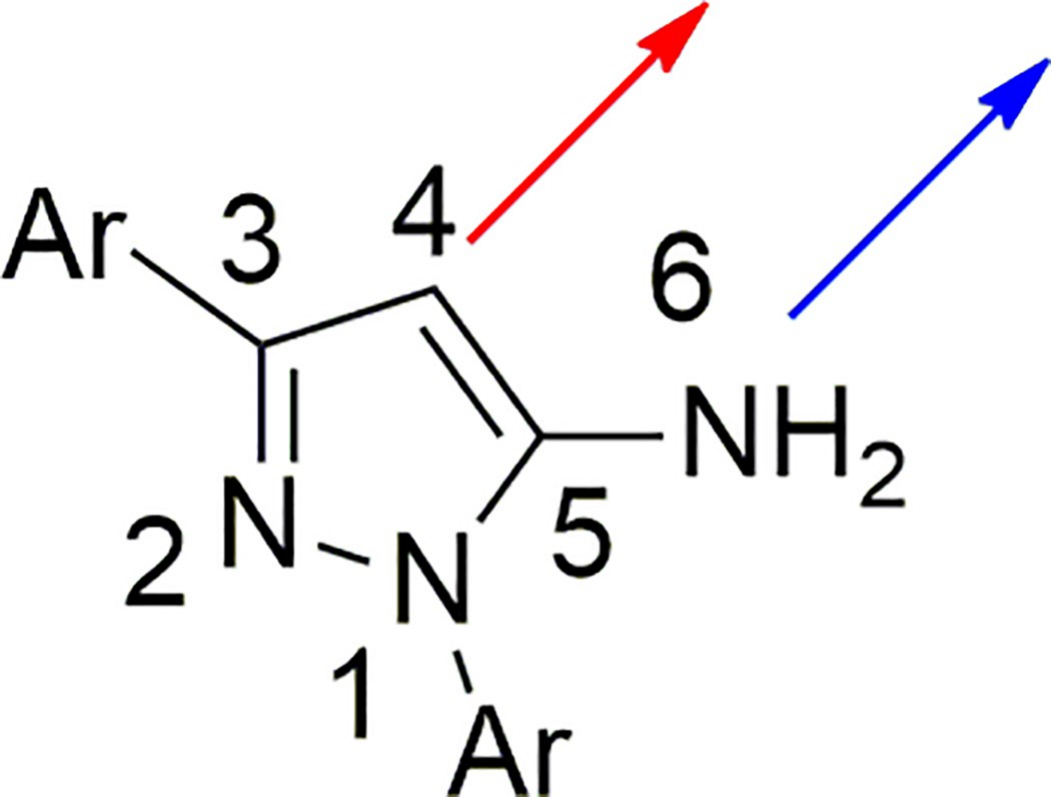
Competitive arylation of 5-aminopyrazoles.

Because of their various characteristic and uses, aryl-substituted 5-aminopyrazoles at C-4 position have recently garnered a lot of interest [32–35]. However, the C-4 arylation of 5-aminopyrazoles can be challenging because of the presence of NH_2_ group that facilitates the competitive N-6 arylation (Fig 1). This can lead to undesired side reaction thus lowering the yield of the desired product [32]. Therefore, controlling the selectivity of the reaction in favor of C-4 arylation is crucial. Furthermore, the presence of bulky groups on either the arylating reagent or 5-aminopyrazole may result in lower reactivity of C-4 position in the reaction [36].

**Fig 2:**
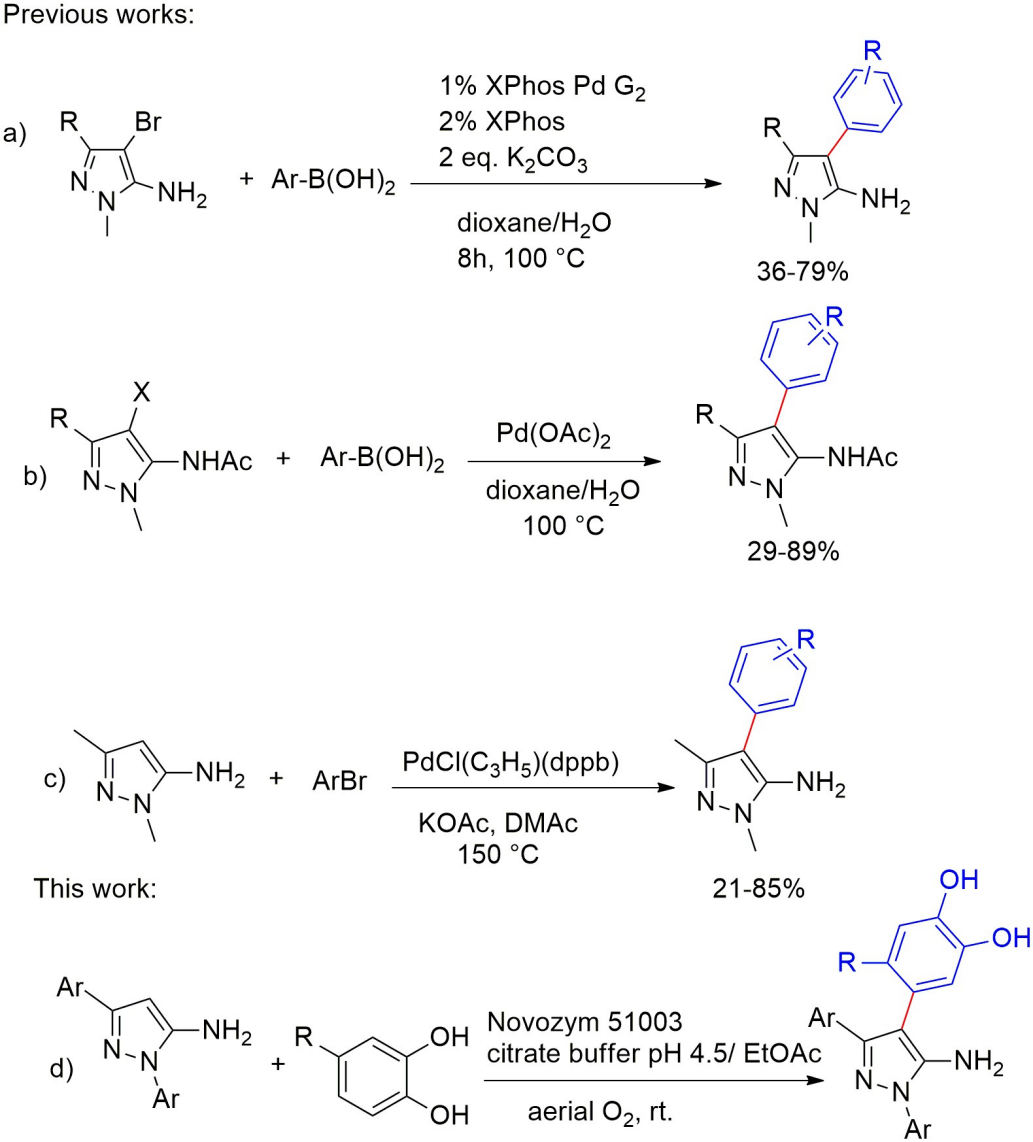
Previous works and this work.

To overcome these challenges, halogenation of the C-4 position followed by performing the Suzuki-Miura cross-coupling reaction has been proposed [32] (Fig 2a). As an alternative for cross-coupling, the simultaneous protection of the amine group and halogenation of the target carbon has been adopted [32] (Fig 2b). Direct arylation has also been used to introduce an aryl group onto the C-4 position, bypassing the amine protection of 5-aminopyrazoles or pre-functionalization of the arylating reagent. However, performing the reaction in high temperature and necessity of using toxic solvents such as dioxane are some drawbacks of this approach [36] (Fig 2c). We here report a novel laccase-catalyzed strategy for the chemoselective arylation of 5-aminopyrazoles at C-4 position under extremely mild condition without the need for pre-activation of the target carbon or protecting the amine group (Fig 2d).

Laccases are multi-copper oxidases whose active site consists of four copper centers and are classified into three groups: type 1 (one copper, T1), type 2 (one copper, T2), and type 3 (two copper, T3) [37]. Laccases use aerial oxygen as an oxidant and produce water as the only byproduct, which is important in terms of green chemistry. These enzymes have various applications such as bioremediation [38], biosensors [39], textiles [40], food [41], and synthesis of organic compounds [42]. Laccases catalyzed the synthesis of various compounds through the oxidation of, for example, phenols (catechols) to their active intermediates (orthoquinons) which generally have a redox potential in the range of laccases [37]. The laccase-catalyzed synthesis of benzofurans [43], benzothia-zoles [44], and functionalization of C-H bonds [45, 46] have been previously well-documented. Following the recent studies conducted by our group to implement laccases as green catalysts in the synthesis of organic compounds [47, 48], enzymatic arylation of 5-aminopyrazoles is presented here for the first time.

## Materials and methods

### General remarks

All reagents are commercially available and used without further purification. Solvents used for extraction and purification were distilled before use. *Myceliophthora thermophila* laccase (Novozym 51003) was a generous gift from Novozymes (Copenhagen, Denmark). Reactions were monitored by thin-layer chromatography (TLC) using silica gel 60 F_254_. All organic synthesis products were purified by preparative thin-layer chromatography (TLC), (CAMAG^®^ instrument, in-house prepared 20 × 20 cm silica plates) and characterized by NMR spectroscopy. ^1^H and ^13^C NMR spectra were recorded at 300 (75) MHz on a Bruker Avance spectrometer using DMSO-*d6* and CDCl_3_ as solvents. The chemical shifts were referenced to the solvent signals at δH/C 2.49/39.50 ppm (DMSO-*d6*) and δH/C 7.26/77 ppm (CDCl_3_) relative to TMS. Melting points were determined with a Thermo Scientific 9100 melting point apparatus and are uncorrected. Mass spectra were recorded with an Agilent Technologies (HP) 5973 mass spectrometer.

### Synthesis of α-bromoketones

15 mmol of the corresponding methyl ketone was dissolved in 10 mL of glacial acetic acid and 18 mmol of bromine solution was added dropwise to the reaction medium at room temperature. After the consumption of starting materials, the reaction mixture was poured into ice and the precipitate was filtered, washed with water, and dried at room temperature.

### Synthesis of α-cyanoketones

α-cyanoketones were prepared according to literature [49]: 10 mmol of the prepared α-bromoketone was dissolved in a mixture of water and ethanol with a ratio of 1:5 and stirred in an ice water bath. Then 30 mmol of sodium cyanide was added to the reaction mixture and stirred for 16 h at room temperature. After the completion of the reaction monitored by thin layer chromatography, 5 mL of water was added to the reaction mixture and filtered. Then, 8 mL of concentrated hydrochloric acid was added to the filtrate to remove excess sodium cyanide (this is done due to the release of hydrogen cyanide gas under fume hood). After the complete removal of hydrogen cyanide gas, the resulting mixture was extracted three times with ethyl acetate. The organic phase was dried under reduced pressure and obtained precipitate dried at room temperature.

### Synthesis of 5-aminopyrazoles

**Fig 3:**
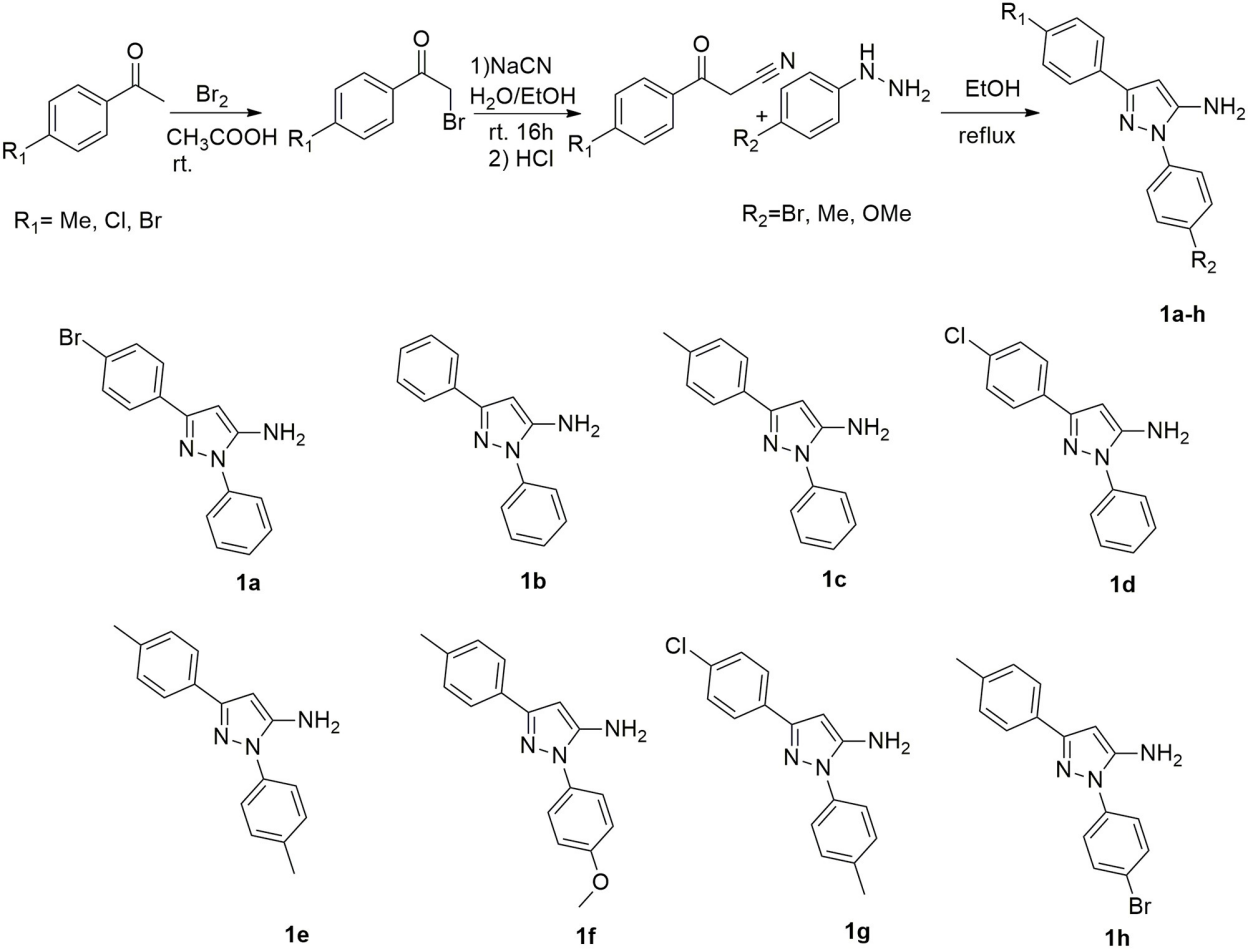
Synthesis of 5-aminopyrazoles 1a-1h.

5 mmol of cyanoaketone prepared in the previous step and 5.6 mmol of phenylhydrazine hydrochloride were dissolved in 15 mL of ethanol and refluxed for 12 h. After ensuring the completion of the starting materials, the solvent was minimized under reduced pressure, then the reaction mixture was poured into ice water and the precipitate was filtered and dried at room temperature (Fig 3). Selected 5-aminopyrazoles (**1a, 1b, 1c, 1d**) were characterized by ^1^H NMR spectroscopy to confirm their structure and purity.

### General procedure for synthesis 3a-j

A 100 mL round bottom flask with a magnetic stirrer bar was charged with a solution of 0.1 mmol of the corresponding 5-aminopyrazole, 0.15 mmol of catechol, 8 mL of 0.01 M citrate buffer pH 4.5, 4 mL of ethyl acetate and *Myceliophthora thermophila* laccase (1 mL) (1000 U) and the mixture was stirred under air. The reaction was monitored with TLC until it was completely consumed. Then the reaction mixture was diluted with EtOAc, the layers were separated and the aqueous phase was extracted with EtOAc (3 x 20 mL). The combined organic phases were dried with anhydrous sodium sulfate, and filtered, and the solvent was removed under reduced pressure. The reaction mixture was purified by preparative TLC (eluting with *n*-hexane/ ethyl acetate = 5/1 to 2/1), provided target compound 3.

## Results

**3-(4-bromophenyl)-1-phenyl-1H-pyrazol-5-amine (1a)** cream solid, isolated yield = 85%, ^1^H NMR (300 MHz, DMSO-*d*_6_) δ 7.5–7.9 (m, 8H), 7.37 (m, 1H), 6.0 (s, 1H), 5.5 (s, 2H).

**1,3-diphenyl-1H-pyrazol-5-amine (1b)** brown solid, isolated, yield = 97% ^1^H NMR (300 MHz, Chloroform-*d*) δ 7.8 (d, *J* = 7.5 Hz, 2H), 7.6 (d, *J* = 7.8 Hz, 2H), 7.5 (t, *J* = 7.9 Hz, 2H), 7.4 (m, 4H), 6.0 (d, *J* = 2.3 Hz, 1H), 4.1 (s, 2H).

**1-phenyl-3-(p-tolyl)-1H-pyrazol-5-amine (1c)** cream solid, isolated yield = 93% ^1^H NMR (300 MHz, Chloroform-*d*) δ 7.7 (d, *J* = 7.6 Hz, 2H), 7.6 (d, *J* = 7.8 Hz, 2H), 7.5 (t, *J* = 7.6 Hz, 2H), 7.3–7.4 (m, 1H), 7.2 (d, *J* = 7.7 Hz, 2H), 6.0 (s, 1H), 4.4 (s, 2H), 2.4 (s, 3H).

**3-(4-chlorophenyl)-1-phenyl-1H-pyrazol-5-amine (1d)** cream solid, isolated yield = 83% ^1^H NMR (300 MHz, DMSO-d_6_) δ 7.7 (m, 5H), 7.3–7.6 (m, 4H), 5.9 (s, 1H), 5.5 (s, 2H).

**4-(5-amino-3-(4-bromophenyl)-1-phenyl-1H-pyrazol-4-yl)benzene-1,2-diol (3a)** brown solid, melting point: 170–172°C, isolated yield = 62%, ^1^H NMR (300 MHz, DMSO-*d*_6_) δ 9.0 (s, 2H), 7.7 (d, *J* = 7.9 Hz, 2H), 7.5 (m, 4H), 7.3–7.4 (m, 3H), 6.8 (d, *J* = 8.0 Hz, 1H), 6.6 (d, *J* = 2.1 Hz, 1H), 6.5 (dd, *J* = 8.0, 2.1 Hz, 1H), 4.9 (s, 2H). ^13^C NMR (75 MHz, DMSO-*d*_6_) δ 147.2, 145.9, 145.0, 144.6, 139.5, 133.5, 131.5, 129.64, 124.0, 123.6, 121.2, 120.9, 117.6, 116.6, 104.1. MS: (EI, 70 eV): m/z = 423 [M^+^]. Anal. Calcd. for C_21_H_16_BrN_3_O_2_: C, 59.73; H, 3.82; N, 9.95. Found: C, 59.66; H, 3.81; N, 9.87.

**4-(5-amino-1,3-diphenyl-1H-pyrazol-4-yl)benzene-1,2-diol (3b)** brown solid, melting point: 145–147°C, isolated yield = 75%, ^1^H NMR (300 MHz, DMSO-*d*_6_) δ 8.9 (d, 2H), 7.7 (d, *J* = 7.8 Hz, 2H), 7.5 (t, *J* = 7.7 Hz, 2H), 7.4 (d, *J* = 7.0 Hz, 2H), 7.3–7.4 (m, 1H), 7.3 (d, *J* = 7.2 Hz, 3H), 6.7 (d, *J* = 7.6 Hz, 1H), 6.6 (s, 1H), 6.5 (d, *J* = 8.2 Hz, 1H), 4.8 (s, 2H). ^13^C NMR (75 MHz, DMSO-*d*_6_) δ 148.4, 145.8, 144.8, 144.47, 139.6, 134.3, 129.6, 128.4, 127.7, 126.9, 123.4, 121.3, 117.7, 116.5, 104.2. MS: (EI, 70 eV): m/z = 343 [M^+^]. Anal. Calcd. for C_21_H_17_N_3_O_2_: C, 73.45; H, 4.99; N, 12.24. Found: C, 73.51; H, 4.31; N, 12.17.

**4-(5-amino-1-phenyl-3-(p-tolyl)-1H-pyrazol-4-yl)benzene-1,2-diol (3c)** white solid, melting point: 188–190°C, isolated yield = 83%, ^1^H NMR (300 MHz, DMSO-*d*_6_) δ 8.7–9.0 (m, 2H), 7.7–7.8 (m, 2H), 7.5–7.6 (m, 2H), 7.3–7.4 (m, 3H), 7.0–7.2 (m, 2H), 6.7–6.9 (m, 1H), 6.6–6.7 (m, 1H), 6.5 (d, *J* = 9.2 Hz, 1H), 4.8 (s, 2H), 2.3 (s, 3H). ^13^C NMR (75 MHz, DMSO-*d*_*6*_) δ 148.5, 145.8, 144.7, 144.4, 139.7, 136.9, 131.5, 129.6, 129.0, 127.6, 126.8, 124.5, 123.4, 121.3, 117.7, 116.5, 104.1, 21.2. MS: (EI, 70 eV): m/z = 357 [M^+^]. Anal. Calcd. for C_22_H_19_N_3_O_2_: C, 73.93; H, 5.36; N, 11.76. Found: C, 73.96; H, 5.40; N, 11.71.

**4-(5-amino-1,3-diphenyl-1H-pyrazol-4-yl)-5-methylbenzene-1,2-diol (3d)** brown solid, melting point: 145–147°C, isolated yield = 91%, ^1^H NMR (300 MHz, DMSO-*d*_6_) δ 8.7 (d, 2H), 7.7 (d, *J* = 7.8 Hz, 2H), 7.5 (t, *J* = 7.6 Hz, 3H), 7.4 (m, 3H), 7.2–7.3 (m, 2H), 6.7 (s, 1H), 6.5 (s, 1H), 4.7 (s, 2H), 1.9 (s, 3H). ^13^C NMR (75 MHz, DMSO-*d*_6_) δ 148.3, 145.1, 145.0, 143.6, 139.8, 134.7, 129.6, 128.6, 128.5, 127.6, 126.6, 123.5, 123.1, 123.0, 118.9, 118.0, 103.4, 19.5. MS: (EI, 70 eV): m/z = 357 [M^+^]. Anal. Calcd. for C_22_H_19_N_3_O_2_: C, 73.93; H, 5.36; N, 11.76. Found: C, 73.91; H, 5.31; N, 11.67.

**4-(5-amino-1-phenyl-3-(p-tolyl)-1H-pyrazol-4-yl)-5-methylbenzene-1,2-diol (3e)** brown solid, melting point: 198–200°C, isolated yield = 93%, ^1^HNMR (300 MHz, DMSO-*d*_6_) δ 8.7 (d, 2H), 7.7 (d, *J* = 7.7 Hz, 2H), 7.5(t, *J* = 8.0 Hz, 3H), 7.3 (t, *J* = 7.0 Hz, 2H), 7.0 (d, *J* = 7.7 Hz, 2H), 6.7 (s, 1H), 6.5 (s, 1H), 4.7 (s, 2H), 2.2 (s, 3H), 1.9 (s, 3H). ^13^C NMR (75 MHz, DMSO-*d*_6_) δ 148.3, 145.0, 144.9, 143.6, 136.8, 131.88, 129.6, 129.12, 126.6, 123.1, 118.0, 103.3, 21.2, 19.5. MS: (EI, 70 eV): m/z = 371 [M^+^]. Anal. Calcd. for C_23_H_21_N_3_O_2_: C, 74.37; H, 5.70; N, 11.31. Found: C, 74.31; H, 5.72; N, 11.37.

**4-(5-amino-3-(4-chlorophenyl)-1-phenyl-1H-pyrazol-4-yl)benzene-1,2-diol (3f)** brown solid, melting point: 143–145°C, isolated yield = 54%, ^1^H NMR (300 MHz, DMSO-*d*_6_) δ 7.72 (d, *J* = 7.9 Hz, 2H), 7.53 (t, *J* = 7.6 Hz, 2H), 7.45 (d, *J* = 8.1 Hz, 2H), 7.36 (d, *J* = 7.9 Hz, 3H), 6.78 (d, *J* = 8.0 Hz, 1H), 6.61 (s, 1H), 6.50 (d, *J* = 8.1 Hz, 1H). ^13^C NMR (75 MHz, DMSO-*d*_6_) δ 147.2, 146.0, 145, 144.7, 139.5, 133.2, 132.3, 129.6, 129.3, 128.6, 127.0, 123.9, 123.5, 121.2, 117.7, 116.7, 104.1. MS: (EI, 70 eV): m/z = 377 [M^+^]. Anal. Calcd. for C_21_H_16_ClN_3_O_2_: C, 66.76; H, 4.27; N, 11.12. Found: C, 66.71; H, 4.31; N, 11.17.

**4-(5-amino-1-(4-methoxyphenyl)-3-(p-tolyl)-1H-pyrazol-4-yl)benzene-1,2-diol (3g)** pale brown solid, melting point: 145–147°C, isolated yield = 94%, ^1^H NMR (300 MHz, DMSO-*d*_6_) δ 8.70 (d, *J* = 137.2 Hz, 2H), 7.57 (d, *J* = 8.5 Hz, 2H), 7.31 (d, *J* = 7.9 Hz, 2H), 7.06 (d, *J* = 8.3 Hz, 4H), 6.75 (d, *J* = 8.1 Hz, 1H), 6.66–6.53 (m, 1H), 6.53–6.34 (m, 1H), 4.71 (s, 2H), 3.80 (s, 3H), 2.25 (s, 3H). ^13^C NMR (75 MHz, DMSO-*d*_6_) δ 158.2, 147.9, 145.8, 144.5, 144.4, 136.7, 132.6, 131.6, 129.0, 127.6, 125.4, 124.6, 121.2, 117.7, 116.5, 114.7, 103.6, 55.8, 21.2. MS: (EI, 70 eV): m/z = 387 [M^+^]. Anal. Calcd. for C_23_H_21_N_3_O_3_: C, 71.30; H, 5.46; N, 10.85. Found: C, 71.21; H, 5.31; N, 10.77.

**4-(5-amino-1,3-di-p-tolyl-1H-pyrazol-4-yl)benzene-1,2-diol (3h)** pale brown solid, melting point: 165–167°C, isolated yield = 91%, ^1^H NMR (300 MHz, DMSO-*d*_6_) δ 8.99 (s, 2H), 7.60 (d, *J* = 8.0 Hz, 2H), 7.44–7.23 (m, 4H), 7.09 (d, *J* = 7.9 Hz, 2H), 6.77 (d, *J* = 8.0 Hz, 1H), 6.68–6.56 (m, 1H), 6.56–6.43 (m, 1H), 4.79 (s, 2H), 2.38 (s, 3H), 2.28 (s, 3H). ^13^C NMR (75 MHz, DMSO-*d*_6_) δ 148.2, 145.8, 144.5, 144.4, 137.2, 136.8, 136.2, 131.6, 130.03, 129.0, 127.6, 124.6, 123.5, 121.2, 117.7, 116.5, 103.9, 21.3, 21.1. MS: (EI, 70 eV): m/z = 371 [M^+^]. Anal. Calcd. for C_23_H_21_N_3_O_2_: C, 74.37; H, 5.70; N, 11.31. Found: C, 74.41; H, 5.61; N, 11.47.

**4-(5-amino-1-(4-bromophenyl)-3-(p-tolyl)-1H-pyrazol-4-yl)benzene-1,2-diol (3i)** pale brown solid, melting point: 142–144°C, isolated yield = 42%, ^1^H NMR (300 MHz, DMSO-*d*_6_) δ 8.57 (s, 2H), 7.70 (s, 4H), 7.32 (d, *J* = 7.9 Hz, 2H), 7.09 (d, *J* = 7.8 Hz, 2H), 6.75 (d, *J* = 8.1 Hz, 1H), 6.58 (s, 1H), 6.47 (d, *J* = 8.0 Hz, 1H), 4.94 (s, 2H), 2.27 (s, 3H). ^13^C NMR (75 MHz, DMSO-*d*_6_) δ 148.9, 145.8, 144.9, 144.5, 139.0, 137.1, 132.4, 131.2, 129.1, 127.7, 125.2, 124.3, 121.3, 119.2, 117.7, 116.5, 104.5, 21.3. MS: (EI, 70 eV): m/z = 437 [M^+^]. Anal. Calcd. for C_22_H_18_BrN_3_O_2_: C, 60.56; H, 4.16; N, 9.63. Found: C, 60.40; H, 4.11; N, 9.67.

**4-(5-amino-3-(4-chlorophenyl)-1-(p-tolyl)-1H-pyrazol-4-yl)benzene-1,2-diol (3j)** pale brown solid, melting point: 162–164°C, isolated yield = 59%, ^1^H NMR (300 MHz, DMSO-*d*_6_) δ 8.67 (s, 2H), 7.59 (d, *J* = 8.0 Hz, 2H), 7.50–7.39 (m, 2H), 7.34 (dd, *J* = 8.3, 5.5 Hz, 4H), 6.78 (d, *J* = 7.9 Hz, 1H), 6.61 (s, 1H), 6.49 (d, *J* = 8.0 Hz, 1H), 4.85 (s, 2H), 2.38 (s, 3H). ^13^C NMR (75 MHz, DMSO-*d*_6_) δ 146.9, 145.9, 144.9, 144.6, 137.0, 136.4, 133.3, 132.23, 130.1, 129.3, 128.5, 124.1, 123.6, 121.2, 117.6, 116.6, 103.9, 21.1. MS: (EI, 70 eV): m/z = 391 [M^+^]. Anal. Calcd. for C_22_H_18_ClN_3_O_2_: C, 67.43; H, 4.63; N, 10.72. Found: C, 67.40; H, 4.61; N, 10.69.

## Discussion

**Fig 4:**
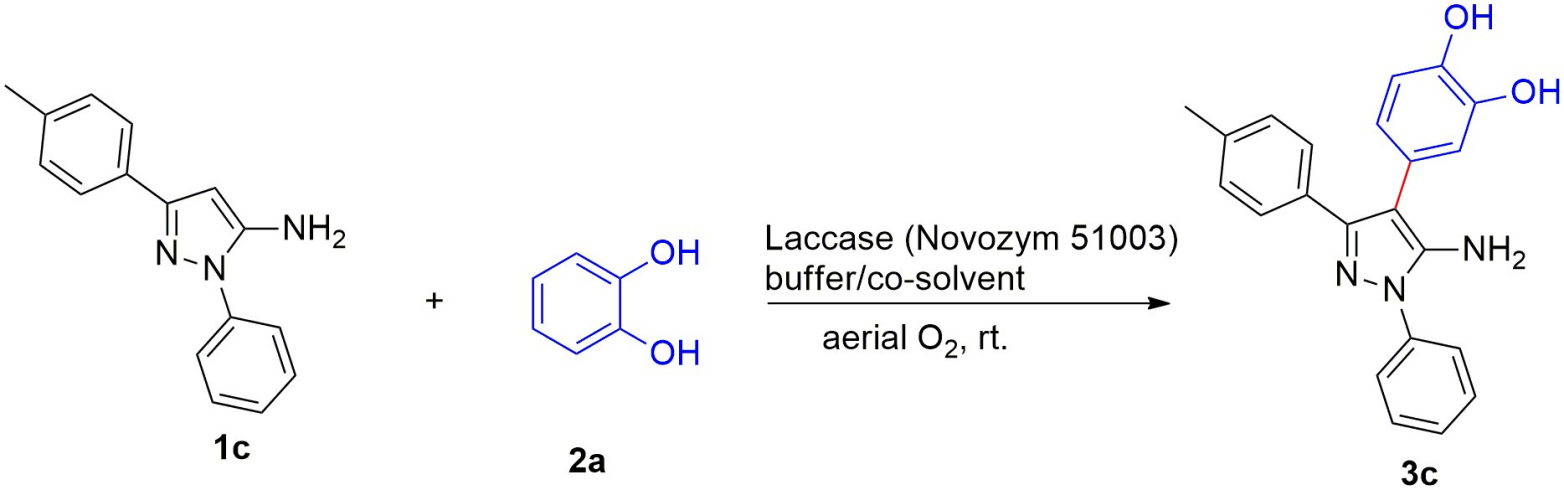
The model reaction.

For the arylation of 5-aminopyrazole in C-4 position, the reaction between 5-amniopyrazole **1c** and catechol **2a** as a model reaction was firstly investigated (Fig 4). The first reaction was performed in phosphate buffer (pH 8, 10 mM) as a solvent and acetonitrile (2:1) as a co-solvent in the presence of laccase (Novozyme 51003^®^). No detectable product was observed after 24 h of the reaction (Table 1 entry 1). By replacing the co-solvent with ethyl acetate (2:1), the desired product was obtained with a yield of 55% shortly after 4 h (Table 1 entry 2) probably due to the better solubility of the 5-aminopyrazole in ethyl acetate. To increase the reaction efficiency, the solvent was changed to citrate buffer (pH 4.5, 10 mM). The results showed that lowering the pH caused to improve the yield to 89% within 4 h (Table 1 entry 3). As reported in previous studies, the reason for this is attributed to higher fungal laccase activity in acidic pH compared to basic pH. By further changing the co-solvent to ethanol and acetonitrile in the presence of citrate buffer as a solvent, no product was formed (Table 1 entries 4 and 5). Also by altering the amount of enzyme to 500 U, the reaction efficiency was effectively decreased to 48% (Table 1 entry 6) probably due to the lower concentration of ortho-quinone produced in the presence of a lower amount of enzyme. Furthermore, running a control reaction in the absence of the enzyme showed no product formation, clearly proving the catalytic function of laccase in the reaction (Table 1 entry 7). Performing the arylation of 5-aminopyrazole in citrate buffer (pH 4.5, 10 mM) as a solvent and ethyl acetate as a co-solvent within 4h was found to be the optimal condition of the reaction (Table 1 entry 3).

**Table 1:** Optimization study.

Entry^a^	Enzyme (U)	Solvent system	Time (h)	Isolated yield (%)
1	1000^b^	MeCN/ phosphate buffer pH 8, 10 mM (1:2)	24	0
2	1000	EtOAc/ phosphate buffer pH 8, 10 mM (1:2)	4	55
3	1000	EtOAc/citrate buffer pH 4.5, 10 mM (1:2)	4	89
4	1000	EtOH/citrate buffer pH 4.5, 10 mM (1:2)	24	N.R.
5	1000	MeCN/citrate buffer pH 4.5, 10 mM (1:2)	24	N.R.
6	500	EtOAc/citrate buffer pH 4.5, 10 mM (1:2)	12	48
7	0	EtOAc/citrate buffer pH 4.5, 10 mM (1:2)	24	N.R.
8	1000	EtOAc /phosphate buffer pH 7, 10 mM (1:2)	8	21
9	1000	MeCN/phosphate buffer pH 7, 10 mM (1:2)	24	N.R.

^a^Reaction conditions: **1c** (0.1 mmol), **2a** (0.15 mmol). ^b^The laccase activity as given by the supplier. N.R.: no reaction

**Fig 5:**
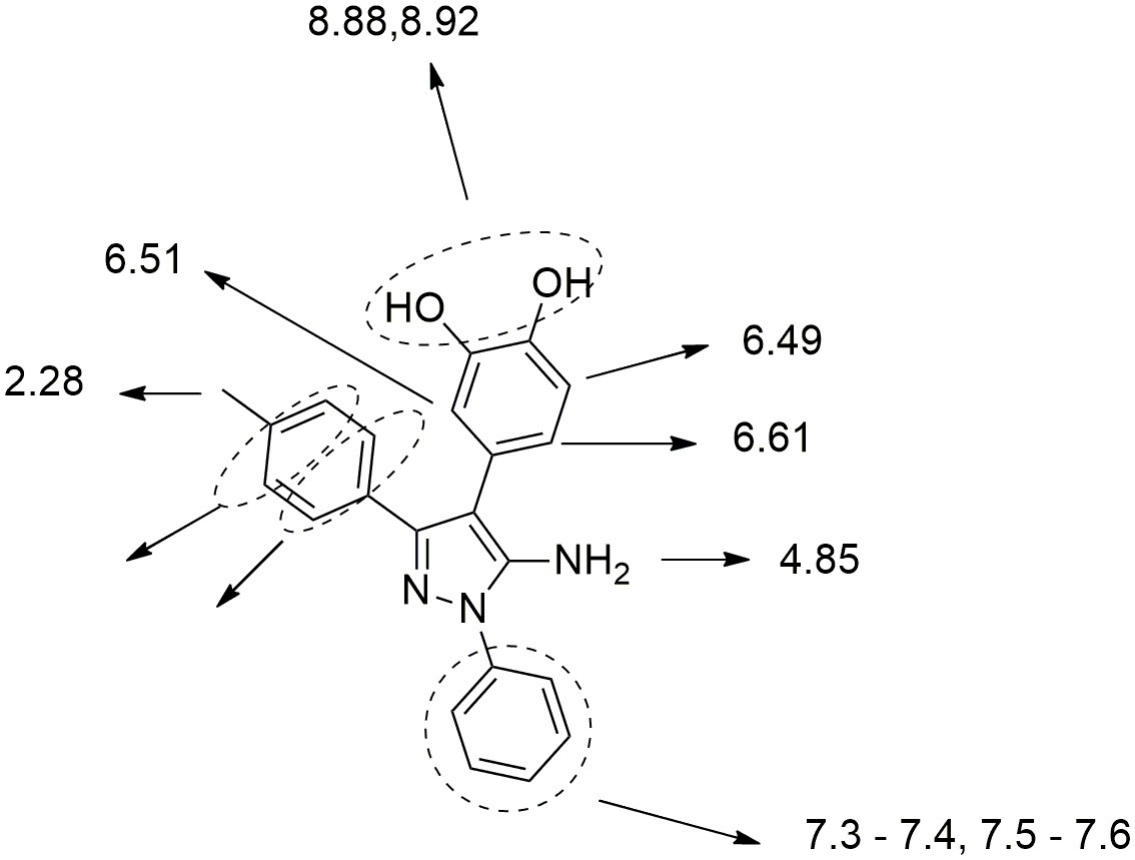
Chemical shifts of 3c in ^1^HNMR.

^1^HNMR, ^13^CNMR and Mass spectrometry was used to prove the chemical structure of the products. The ^1^HNMR spectrum of **3c** as a typical product of the enzymatic reaction showed a singlet with integration of 3 in 2.28 ppm, corresponding to the methyl group of the phenyl ring. The singlet peak at 4.85 ppm with integration of 2 corresponds to the amine group, clearly proving that the reaction is performed via nucleophilic attack of C4 to the ortho-quinone ring. Three peaks with the total integration of 3 at 6.49, 6.51 and 6.61 ppm correspond to catechol hydrogens. The rest of the peaks in aromatic area with integration of 9 can be attributed to the 2 remaining phenyl ringsin the structure. Two singlet peaks at 8.88 and 8.92 correspond to two hydroxyl groups. Compared to the spectrum of the corresponding starting material **1c**, the singlet peak at 5.98 ppm was removed in the product, and the singlet peak at 4.38 ppm remained intact, which indicates the binding of catechol to the carbon of position 4 (Fig 5). In ^13^CNMR spectrum of **3c**, 18 peaks were observed while the desired compound has 22 carbon atoms. The increase in the height of some peaks in the aromatic region can be considered as an evidence for theoverlaping of some signals togatherof some carbons. The mass spectrum of the product **3c** further proved its structure by showing the molecular ion (M^+^) of 357, which belongs to the molecular mass of this product.

**Fig 6:**
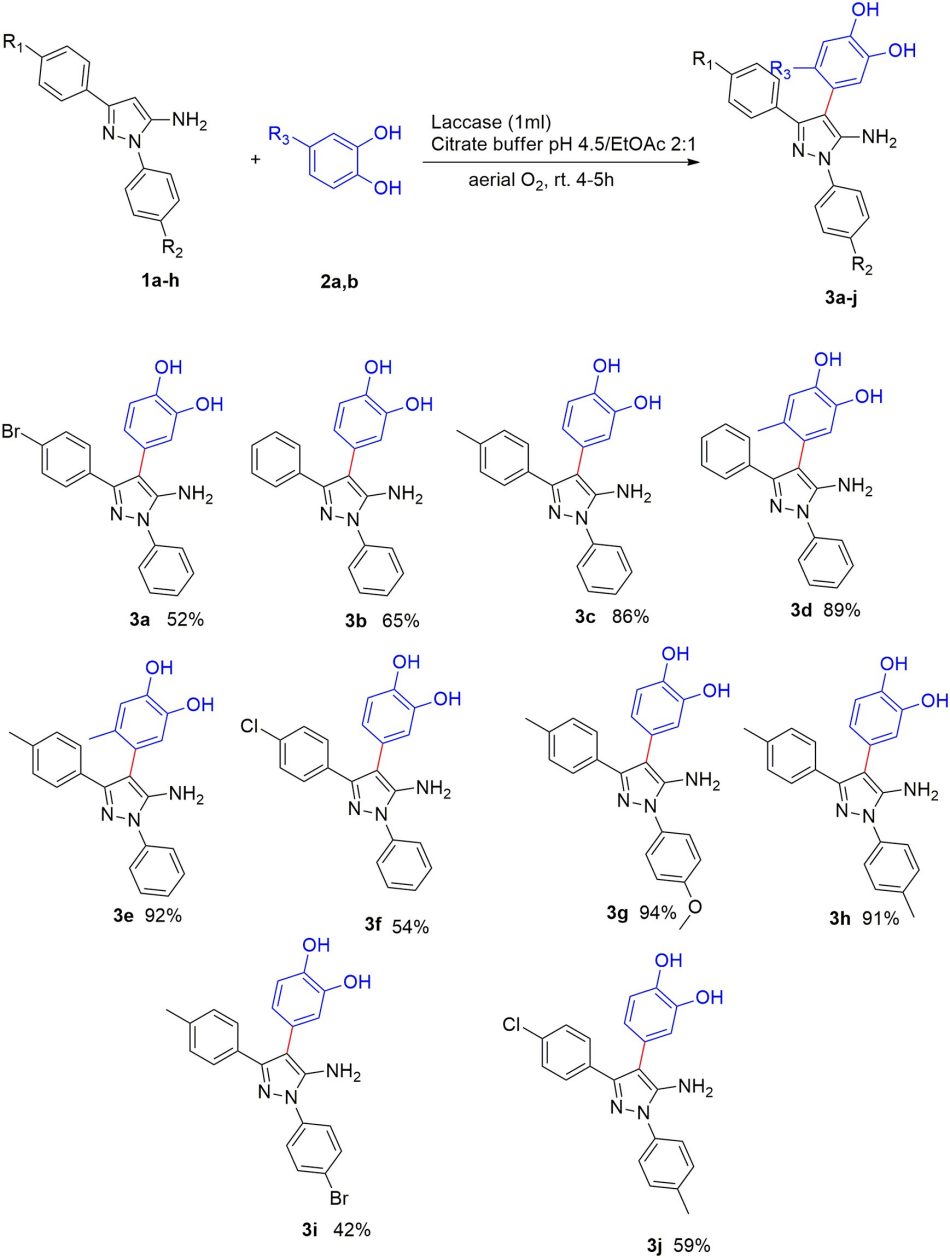
Scope of reaction (Reaction conditions: Laccase (1000 U), 1 (0.1 mmol), 2 (0.15 mmol)).

The optimal conditions were then applied toexpand the scope of the reaction to the substrates with different substitutions (Fig 6). The results showed that when the electron-withdrawing substituent is placed on the 5-aminopyrazole ring, the reaction efficiency decreases (**3a**, **3f**, **3i**, and **3j**), which can be attributed to the decrease in the nucleophilicity of 5-aminopyrazole. When nitrogen number 1 and carbon number 3 simultaneously had electron-donating substituents, the efficiency was associated with an increase (**3g**, **3h**). In addition, the presence of methyl group on catechol ring at C-4 position facilitated the reaction toward higher efficiencies compared those performed with non-substituted catechol (**3b** compared to **3d** and **3c** compared to **3e**).

**Fig 7:**
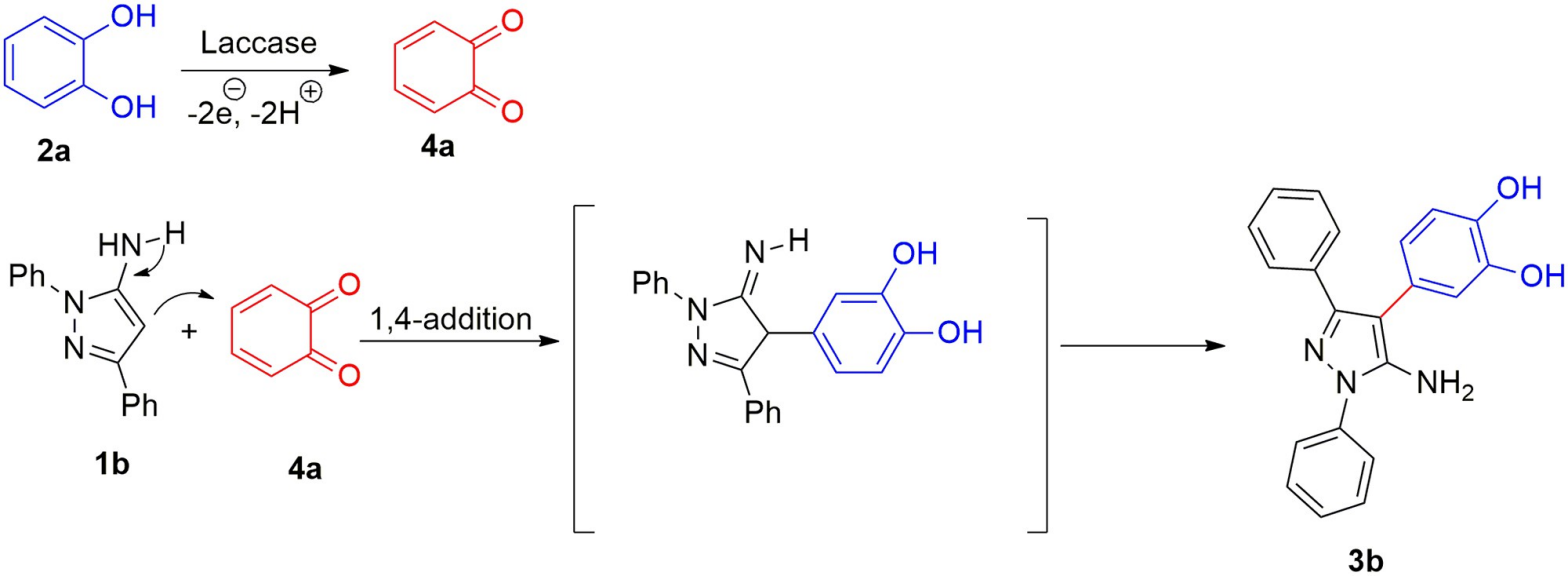
Proposed mechanism.

The possible mechanism for the reaction was proposed based on the control experiments and the previous similar reports [37, 39] on the mechanism of oxidative reactions catalyzed by laccases (Fig 7). The reaction goes through laccase-catalyzed oxidation of the catechol **2a** to the ortho-quinone **4a**. Then 5-aminopyrazole **1a** attacks the quinone intermediate via imine-enamine tautomerization to afford the corresponding product **3b**.

## Conclusions

In this research, for the chemoselective arylation of 5-aminopyrazoles in the C-4 position was introduced. This enzymatic route offered a simple and efficient method for the arylation reaction without prior protection of the amine group. The reaction was carried out in mild conditions without needing any toxic reagents which provides a safe approach in the synthesis of some heterocyclic medicinal compounds.

## Supporting information

S1 File ^1^H, ^13^C NMR and mass spectrum of all synthesized derivatives. (DOCX)

## References

[1] Nishanth Rao R, Jena S, Mukherjee M, Maiti B, Chanda K. Green synthesis of biologically active heterocycles of medicinal importance: A review. Environmental Chemistry Letters, 2021;19: 3315–3358.

[2] Khandelwal S, Tailor YK, Rushell E, Kumar M. Use of sustainable organic transformations in the construction of heterocyclic scaffolds. In: Green Approaches in Medicinal Chemistry for Sustainable Drug Design. 2020; 245–352.

[3] He H, Wan Q, Hou ZW, Zhou Q, Wang L. Organoelectrophotocatalytic generation of acyl radicals from formamides and aldehydes: access to acylated 3-CF3-2-oxindoles. Organic Letters. 2023; 25: 7014–7019. doi: 10.1021/acs.orglett.3c02607 37721400

[4] Zhang Z, Zhang W, Hou ZW, Li P, Wang L. Electrophilic halospirocyclization of N-benzylacrylamides to access 4-halomethyl-2-azaspiro [4.5] decanes. The Journal of Organic Chemistry. 2023; 88: 13610–13621. doi: 10.1021/acs.joc.3c01315 37694951

[5] Faisal M, Saeed A, Hussain S, Dar P, Larik FA. Recent developments in synthetic chemistry and biological activities of pyrazole derivatives. Journal of Chemical Sciences. 2019;131:1–30.

[6] Fustero S, Simon-Fuentes A, Sanz-Cervera JF. Recent advances in the synthesis of pyrazoles. A review. Organic Preparations and Procedures International. 2009;41:253–90.

[7] Ebenezer O, Shapi M, Tuszynski JA. A review of the recent development in the synthesis and biological evaluations of pyrazole derivatives. Biomedicines. 2022;10:1124. doi: 10.3390/biomedicines10051124 35625859 PMC9139179

[8] González-Pelayo S, López LA. Microwave-Assisted Generation and Capture by Azoles of ortho-Quinone Methide Intermediates under Aqueous Conditions. European Journal of Organic Chemistry, 2017; 40: 6003–6007.

[9] Aggarwal R, Kumar V, Kumar R, Singh SP. Approaches towards the synthesis of 5-aminopyrazoles. Beilstein journal of organic chemistry. 2011;7:179–97. doi: 10.3762/bjoc.7.25 21448263 PMC3063075

[10] Shaabani A, Nazeri MT, Afshari R. 5-Amino-pyrazoles: potent reagents in organic and medicinal synthesis. Molecular diversity. 2019;23:751–807. doi: 10.1007/s11030-018-9902-8 30552550

[11] Thore SN, Gupta SV, Baheti KG. Novel ethyl-5-amino-3-methylthio-1H-pyrazole-4-carboxylates: Synthesis and pharmacological activity. Journal of Saudi Chemical Society. 2016;20:259–64.

[12] Swanepoel B, Nitulescu GM, Olaru OT, Venables L, van de Venter M. Anti-cancer activity of a 5-aminopyrazole derivative lead compound (BC-7) and potential synergistic cytotoxicity with cisplatin against human cervical cancer cells. International Journal of Molecular Sciences. 2019;20:5559. doi: 10.3390/ijms20225559 31703393 PMC6888365

[13] Abdel-Aal MT, Abdel-Aleem AA, Ibahim LI, Zein AL. Synthesis and antimicrobial activity of novel 5-amino-4-cyano-1H-pyrazole and quinazolin-4 (3H)-one derivatives. Archives of pharmacal research. 2010;33:1891–900. doi: 10.1007/s12272-010-1202-5 21191752

[14] Praveena G, Yagnam S, Banoth L, Trivedi R, Prakasham R. S. Bacterial biosynthesis of nanosilver: a green catalyst for the synthesis of (amino pyrazolo)-(phenyl) methyl naphth-2-ol derivatives and their antimicrobial potential. New Journal of Chemistry. 2020; 44: 13046–13061.

[15] Marinozzi M, Marcelli G, Carotti A. N-Aryl-5-aminopyrazole: a versatile architecture in medicinal chemistry. Mini reviews in medicinal chemistry. 2015;15:272–99. doi: 10.2174/1389557515666150312154536 25764318

[16] Hebishy AM, Salama HT, Elgemeie GH. New route to the synthesis of Benzamide-Based 5-aminopyrazoles and their fused heterocycles showing remarkable antiavian influenza virus activity. ACS omega. 2020;5:25104–12. doi: 10.1021/acsomega.0c02675 33043189 PMC7542596

[17] Helal MH, Salem MA, Aly HM. Synthesis, Antimicrobial Activity and Molecular Modeling of Some Novel 5-Aminopyrazole, Pyrazolo [1, 5-a] pyrimidine, Bispyrazole and Bispyridone Derivatives Containing Antipyrinyl Moiety. Journal of Heterocyclic Chemistry. 2017;54:2614–26.

[18] Chen J, Liu W, Ma J, Xu H, Wu J, Tang X, et al. Synthesis and properties of fluorescence dyes: tetracyclic pyrazolo [3, 4-b] pyridine-based coumarin chromophores with intramolecular charge transfer character. The Journal of Organic Chemistry. 2012;77:3475–82. doi: 10.1021/jo3002722 22428730

[19] Lin W, Cai Q, Zheng C, Zheng Y, Shi D. Synthesis of functionalized coumarino [4, 3-d] pyrazolo [3, 4-b] pyridine derivatives and their selective recognition for Zn2+. Chinese Journal of Organic Chemistry. 2017;37:2392.

[20] Udhayasurian R, Sivakumar K. Facile NMI-MsCl mediated synthesis of novel pyrazole derivatives bearing heteroaryl amides as potent antimicrobial agents. Organic Chemistry. 2022 Jan 1(part v).

[21] Gao Q, Tian J, Wen K, Chen C, Yao X, Pang J, et al. Copper-Mediated C4-Benzylations of 5-Aminopyrazoles with 3-Indoleacetic Acids. The Journal of Organic Chemistry. 2023; 88: 6623–6632. doi: 10.1021/acs.joc.2c02972 37166183

[22] Kallmeier F, Fertig R, Irrgang T, Kempe R. Chromium-Catalyzed Alkylation of Amines by Alcohols. Angewandte Chemie International Edition. 2020;59:11789–93. doi: 10.1002/anie.202001704 32187785 PMC7384194

[23] Guo G, Liu J, Wang G, Zhang D, Lu J, Zhao G. Synthesis and biological evaluation of 3-(4-fluorophenyl)-1H-pyrazole derivatives as androgen receptor antagonists. Anti-Cancer Drugs. 2016;27:278–85. doi: 10.1097/CAD.0000000000000322 26633887

[24] Chen H, Wang B, Li P, Yan H, Li G, Huang H, et al. The optimization and characterization of functionalized sulfonamides derived from sulfaphenazole against Mycobacterium tuberculosis with reduced CYP 2C9 inhibition. Bioorganic & Medicinal Chemistry Letters. 2021;40:127924. doi: 10.1016/j.bmcl.2021.127924 33705901

[25] Deka B, Baruah PK, Deb ML. Multi-component synthesis of 3-substituted indoles and their cyclisation to α-carbolines via I 2-promoted intramolecular C2 oxidative amination/aromatisation at room temperature. Organic & Biomolecular Chemistry. 2018; 16: 7806–7810. 30328453 10.1039/c8ob02362j

[26] Arias-Gomez A, Godoy A, Portilla J. Functional pyrazolo [1, 5-a] pyrimidines: Current approaches in synthetic transformations and uses as an antitumor scaffold. Molecules. 2021;26:2708. doi: 10.3390/molecules26092708 34063043 PMC8125733

[27] Chang EC, Chen CY, Wang LY, Huang YY, Yeh MY, Wong FF. Synthesis of 5-arylamino-1-arylpyrazoles from 5-aminopyrazoles with arylhalides via CuI catalyzed Ullman coupling reaction. Tetrahedron. 2013;69:570–6.

[28] Sidhom A, Soulé JF, Doucet H, Allouche F. Reactivity of 5-aminopyrazoles bearing a cyclopropyl group at C3-position in palladium-catalyzed direct C4-arylation. Catalysis Communications. 2018;115:55–8.

[29] Jorda R, Schütznerová E, Cankař P, Brychtová V, Navrátilová J, Kryštof V. Novel arylazopyrazole inhibitors of cyclin-dependent kinases. Bioorganic & Medicinal Chemistry. 2015;23:1975–81. doi: 10.1016/j.bmc.2015.03.025 25835357

[30] Ismail MM, Soliman DH, Sabour R, Farrag AM. Synthesis of new arylazopyrazoles as apoptosis inducers: Candidates to inhibit proliferation of MCF-7 cells. Archiv der Pharmazie. 2021;354:2000214. doi: 10.1002/ardp.202000214 32924168

[31] Goldstein DM, Alfredson T, Bertrand J, Browner MF, Clifford K, Dalrymple SA, et al. Discovery of S-[5-Amino-1-(4-fluorophenyl)-1 H-pyrazol-4-yl]-[3-(2, 3-dihydroxypropoxy) phenyl] methanone (RO3201195), an Orally Bioavailable and Highly Selective Inhibitor of p38 Map Kinase. Journal of medicinal chemistry. 2006;49:1562–75. doi: 10.1021/jm050736c 16509574

[32] Jedinák L, Cankař P. 4-Arylation of N-Acylamino-and Aminopyrazoles by the Suzuki–Miyaura Cross-Coupling Reaction. European Journal of Organic Chemistry. 2016;2016:2013–23.

[33] Tomanová M, Jedinák L, Košař J, Kvapil L, Hradil P, Cankař P. Synthesis of 4-substituted pyrazole-3, 5-diamines via Suzuki–Miyaura coupling and iron-catalyzed reduction. Organic & Biomolecular Chemistry. 2017;15:10200–11. 10.1039/c7ob02373a 29177274

[34] Jedinák L, Zátopková R, Zemánková H, Šustková A, Cankař P. The suzuki–Miyaura cross-coupling reaction of halogenated aminopyrazoles: method development, scope, and mechanism of dehalogenation side reaction. The Journal of Organic Chemistry. 2017;82:157–69. doi: 10.1021/acs.joc.6b02306 27997179

[35] Natarajan P, Kanchithalaivan S, Chatterjee A, Peruncheralathan S. Organocatalytic Chemoselective C4-Benzylation of 5-Aminopyrazoles. Asian Journal of Organic Chemistry. 2024; e202300628.

[36] Derridj F, Roger J, Djebbar S, Doucet H. Catalytic System for Inhibition of Amination-Type Reaction and Palladium-Catalysed Direct Arylation using Non-Protected Pyrazole Derivatives. Advanced Synthesis & Catalysis. 2012;354:747–50.

[37] Bassanini I, Ferrandi EE, Riva S, Monti D. Biocatalysis with laccases: An updated overview. Catalysts. 2020;11:26.

[38] Dong CD, Tiwari A, Anisha GS, Chen CW, Singh A, Haldar D, et al. Laccase: A potential biocatalyst for pollutant degradation. Environmental Pollution. 2023;319:120999. doi: 10.1016/j.envpol.2023.120999 36608728

[39] Rodríguez-Delgado MM, Alemán-Nava GS, Rodríguez-Delgado JM, Dieck-Assad G, Martínez-Chapa SO, Barceló D, et al. Laccase-based biosensors for detection of phenolic compounds. TrAC Trends in Analytical Chemistry. 2015;74:21–45.

[40] Pezzella C, Giacobbe S, Giacobelli VG, Guarino L, Kylic S, Sener M, et al. Green routes towards industrial textile dyeing: A laccase based approach. Journal of Molecular Catalysis B: Enzymatic. 2016;134:274–9.

[41] Mayolo-Deloisa K, González-González M, Rito-Palomares M. Laccases in food industry: Bioprocessing, potential industrial and biotechnological applications. Frontiers in bioengineering and biotechnology. 2020;8:222. doi: 10.3389/fbioe.2020.00222 32266246 PMC7105568

[42] Jayakumar J, Priyadarshini D, Parthasarathy A, Reddy SR. Recent advances in molecular oxygen assisted laccase catalyzed sustainable organic transformations. Asian Journal of Organic Chemistry. 2023;12:e202200564.

[43] Wellington KW, Qwebani-Ogunleye T, Kolesnikova NI, Brady D, de Koning CB. One-pot laccase-catalysed synthesis of 5, 6-dihydroxylated benzo [b] furans and catechol derivatives, and their anticancer activity. Archiv der Pharmazie. 2013;346:266–77. doi: 10.1002/ardp.201200413 23447437

[44] Ghorashi N, Shokri Z, Moradi R, Abdelrasoul A, Rostami A. Aerobic oxidative synthesis of quinazolinones and benzothiazoles in the presence of laccase/DDQ as a bioinspired cooperative catalytic system under mild conditions. RSC advances. 2020;10:14254–61. doi: 10.1039/c9ra10303a 35498453 PMC9051882

[45] Chen YJ, Zhang GY, He YH, Guan Z. Aryl C–H amination initiated by laccase-mediated oxidation of 4-phenylurazole. Catalysis Science & Technology. 2019;9:4216–21.

[46] Suljić S, Mortzfeld FB, Gunne M, Urlacher VB, Pietruszka J. Enhanced Biocatalytic Performance of Bacterial Laccase from Streptomyces sviceus: Application in the Michael Addition Sequence Towards 3-Arylated 4-Oxochromanes. ChemCatChem. 2015;7:1380–5.

[47] Shahedi M, Shahani R, Habibi Z, Yousefi M, Brask J, Minaei-Tehrani A, et al. Diarylation of thiazolopyrimidines by laccase and their in vitro evaluation as antitumor agents. Scientific Reports. 2022;12:22326. doi: 10.1038/s41598-022-26820-9 36567332 PMC9790884

[48] Shahedi M, Omidi N, Habibi Z, Yousefi M, Brask J, Notash B, et al. Biocatalytic stereoselective synthesis of pyrrolidine-2, 3-diones containing all-carbon quaternary stereocenters. Organic & Biomolecular Chemistry. 2023;21:2742–7. doi: 10.1039/d2ob02294j 36916669

[49] Eagon S, Ball-Jones N, Haddenham D, Saavedra J, DeLieto C, Buckman M, et al. Enantioselective reduction of α-substituted ketones mediated by the boronate ester TarB-NO_2_. Tetrahedron Letters. 2010;51(49):6418–21.

