# Supplementary material for: Laccase-mediated chemoselective C-4 arylation of 5-aminopyrazoles
Source: PLoS One. 2024 Sep 18;19(9):e0308036. doi: 10.1371/journal.pone.0308036 (PMC11410246; doi:10.1371/journal.pone.0308036)
Supplement: S1 File — (DOCX) [file pone.0308036.s001.docx]

**Laccase-mediated chemoselective C-4 arylation of 5-aminopyrazoles**

Mansour Shahedi ^1^, Rojina Shahani ^1^, Niloofar Omidi ^1^, Zohreh Habibi ^1^*, Maryam Yousefi ^2^, Mehdi Mohammadi ^3^*

^1^ Department of Organic Chemistry, Shahid Beheshti University, 1983969411 Tehran, Iran

^2^ Nanobiotechnology Research Center, Avicenna Research Institute, ACECR, Tehran, Iran

^3^ Bioprocess Engineering Department, Institute of Industrial and Environmental Biotechnology, National Institute of Genetic Engineering and Biotechnology (NIGEB), Tehran, Iran

Corresponding Authors Email: z_habibi@sbu.ac.ir; [m.mohammadi@nigeb.ac.ir](mailto:m.mohammadi@nigeb.ac.ir)


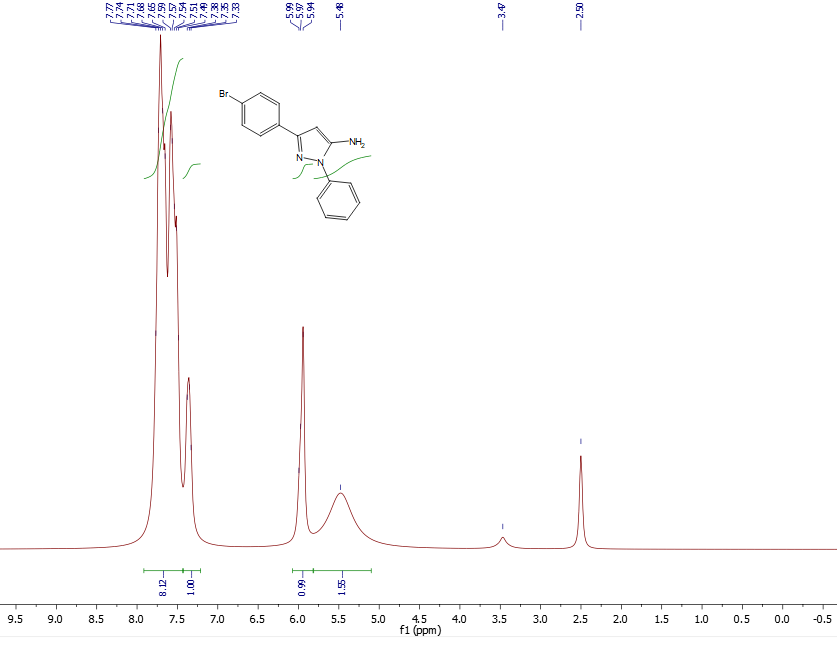


^1^HNMR (300 MHz, DMSO-d_6_) of compound **1a**


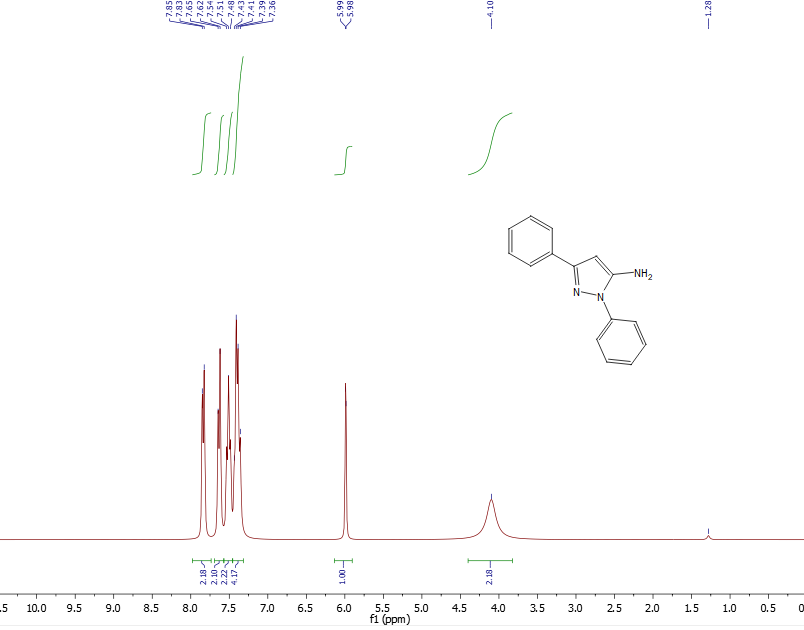
 ^1^HNMR (300 MHz, CDCl_3_) of compound **1b**


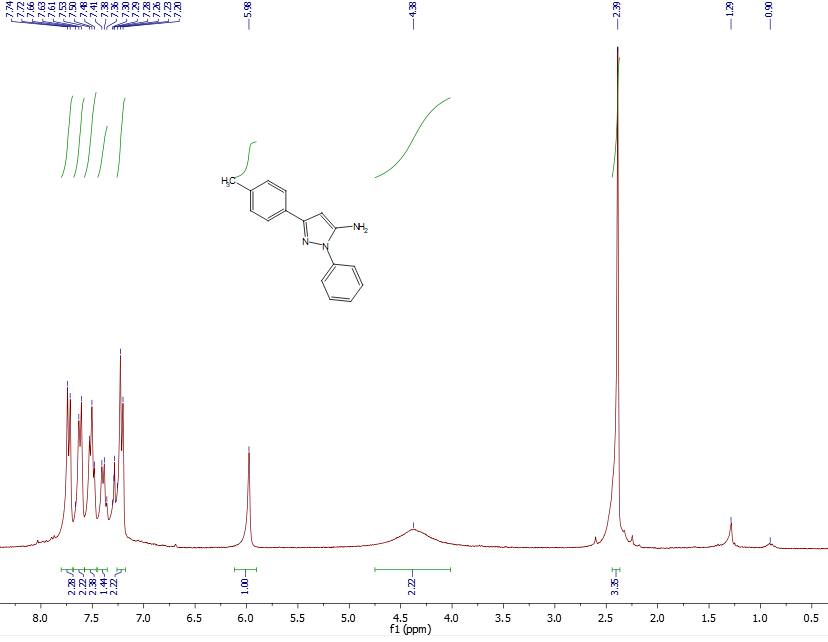


^1^HNMR (300 MHz, CDCl_3_) of compound **1c**


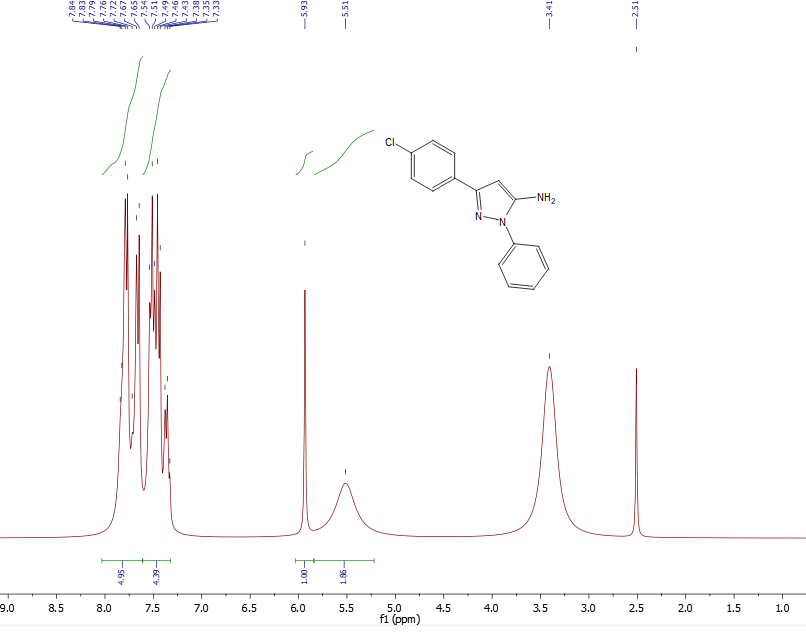


^1^HNMR (300 MHz, DMSO-d_6_) of compound **1d**


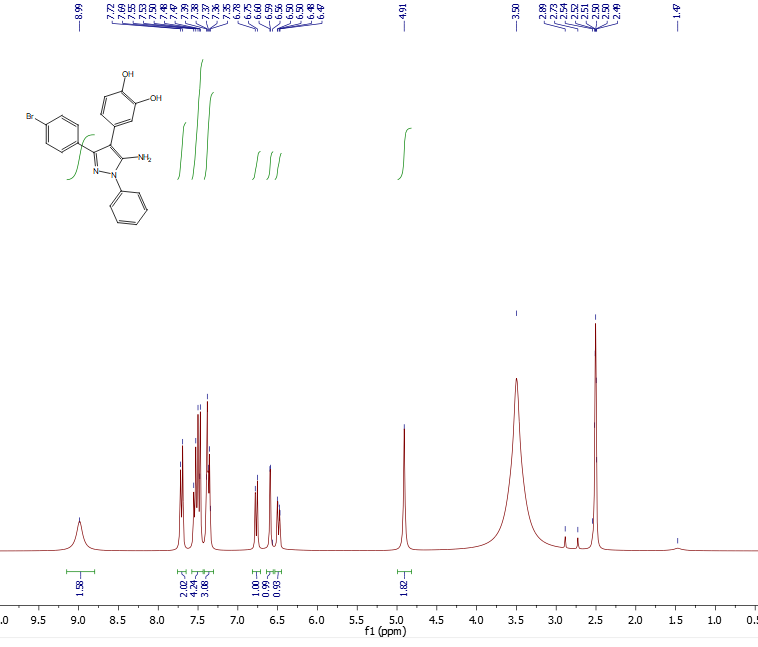


^1^HNMR (300 MHz, DMSO-d_6_) of compound **3a**


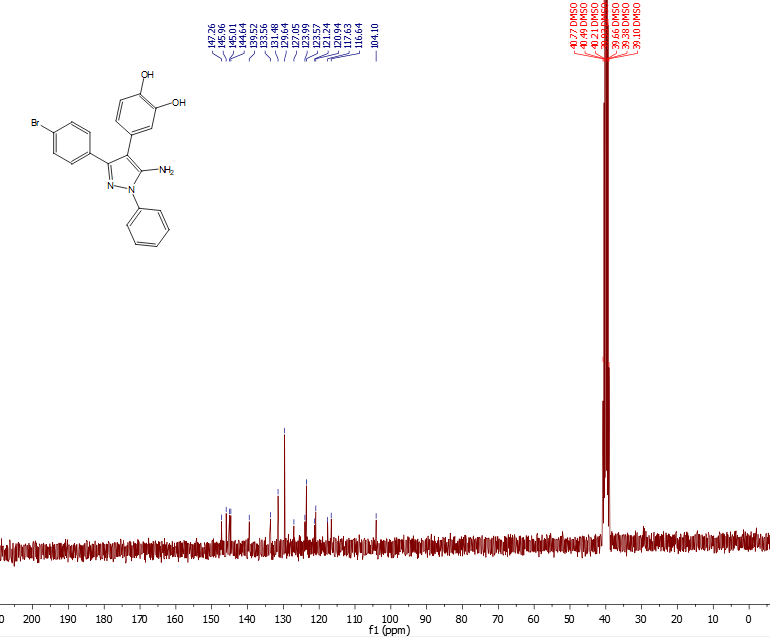


^13^CNMR (75 MHz, DMSO-d_6_) of compound **3a**

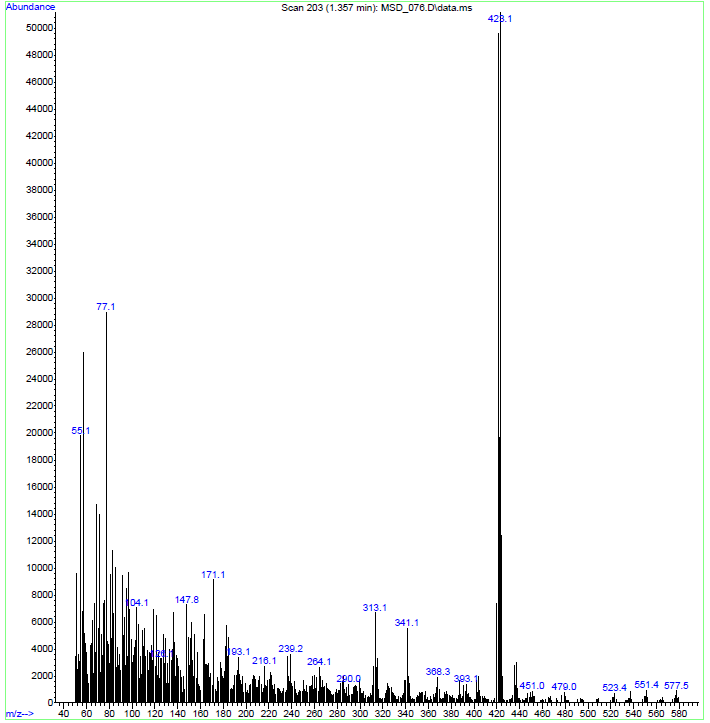


Mass spectra of **3a**


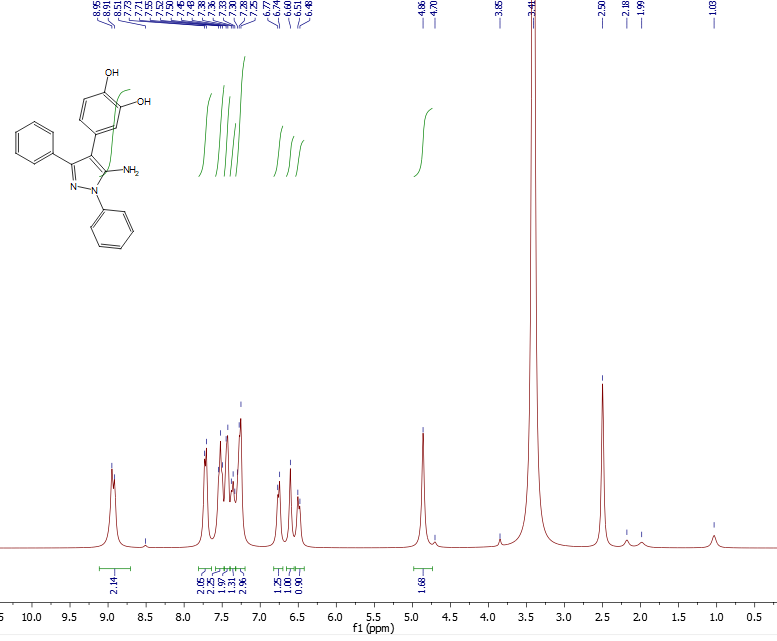


^1^HNMR (300 MHz, DMSO-d_6_) of compound **3b**


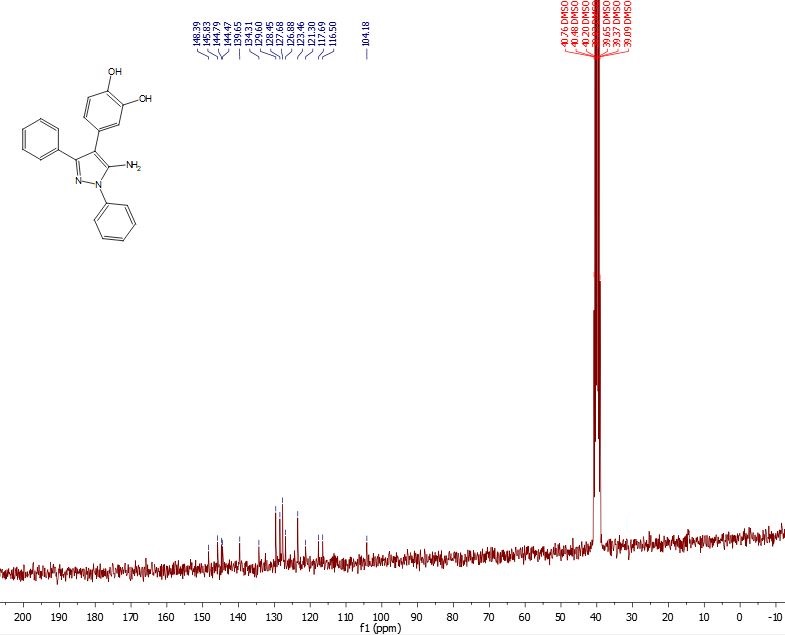


^13^CNMR (75 MHz, DMSO-d_6_) of compound **3b**

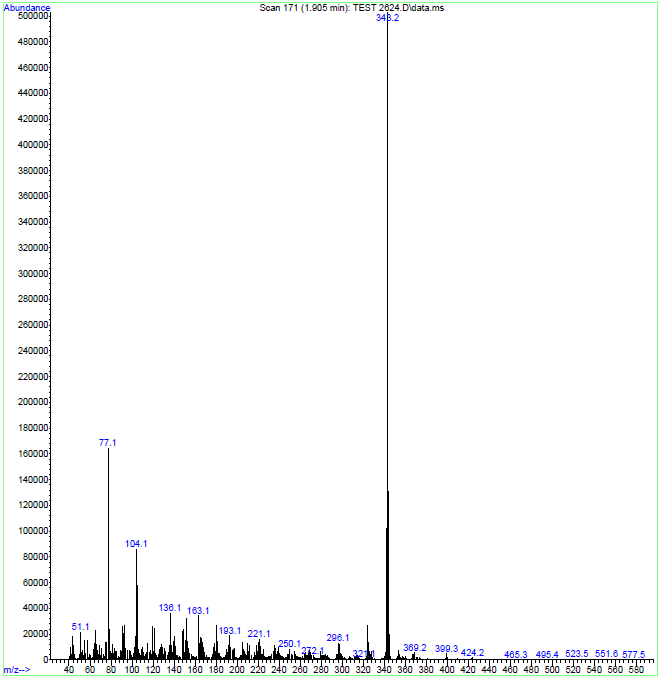


Mass spectra of **3b**


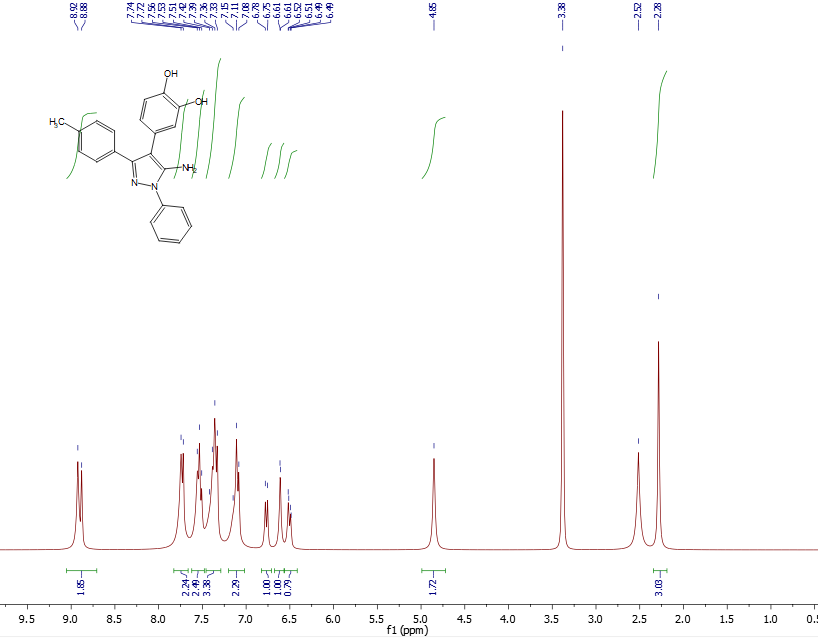


^1^HNMR (300 MHz, DMSO-d_6_) of compound **3c**


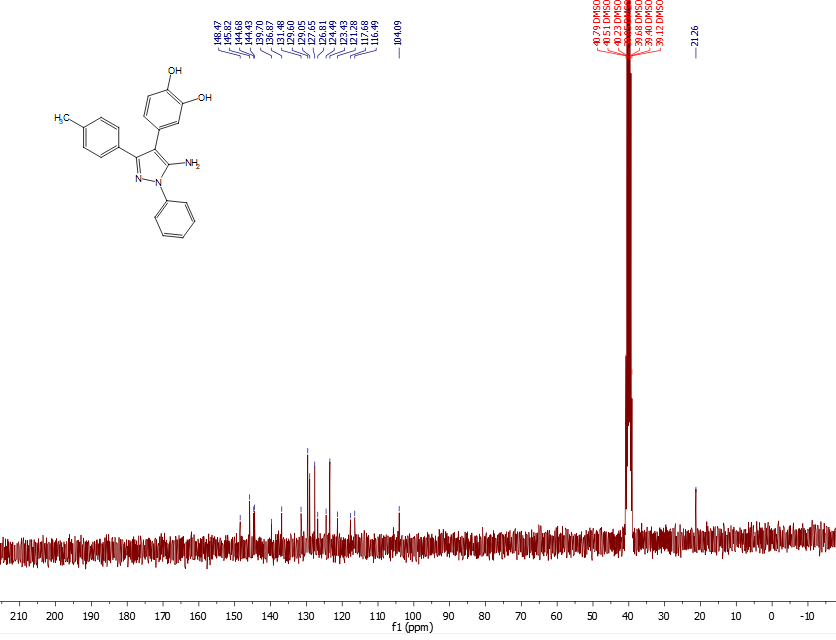


^13^CNMR (75 MHz, DMSO-d_6_) of compound **3c**

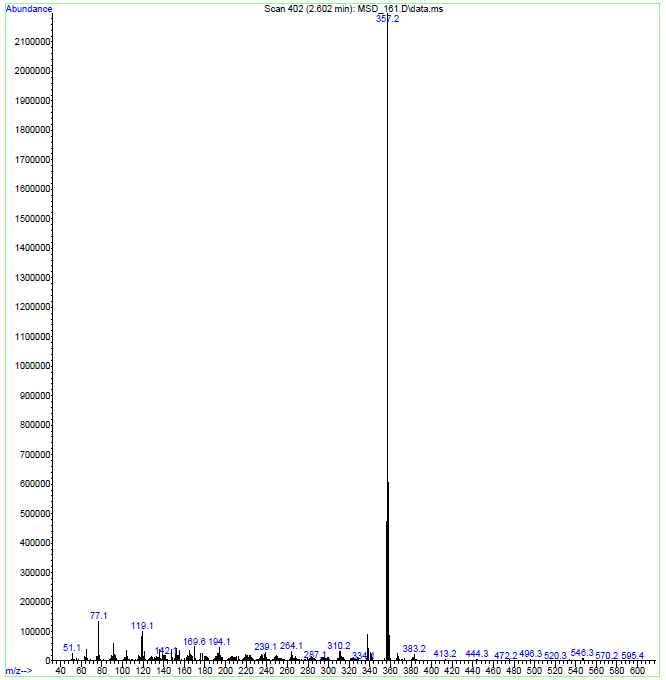


Mass spectra of **3c**


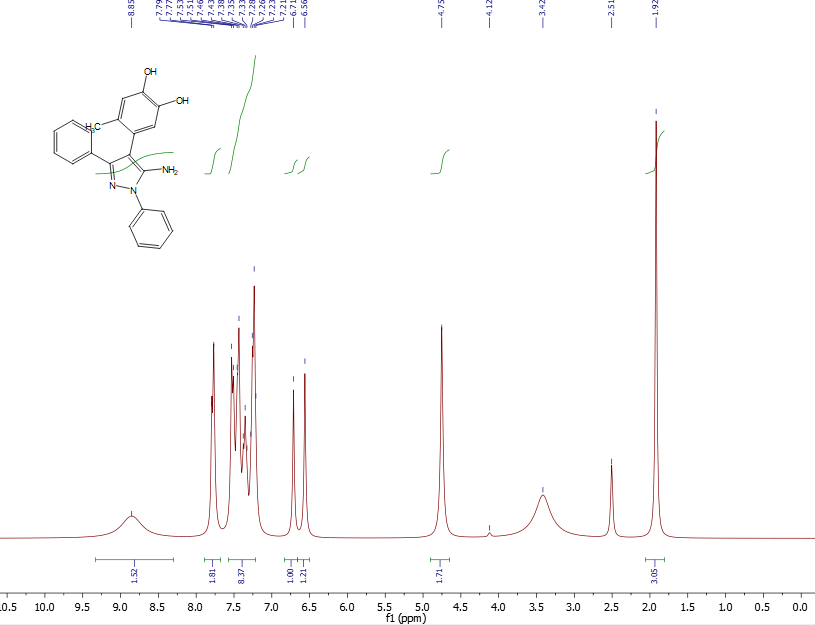


^1^HNMR (300 MHz, DMSO-d_6_) of compound **3d**


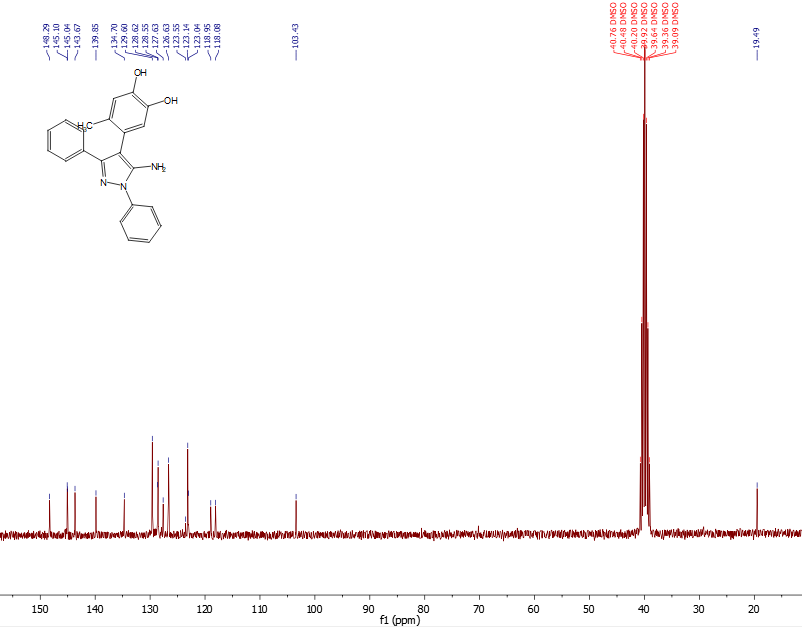


^13^CNMR (75 MHz, DMSO-d_6_) of compound **3d**


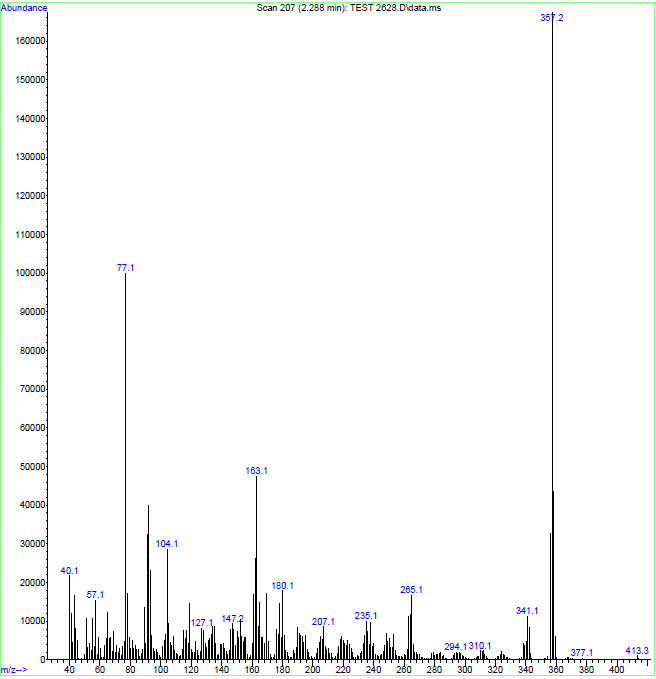


Mass spectra of **3d**


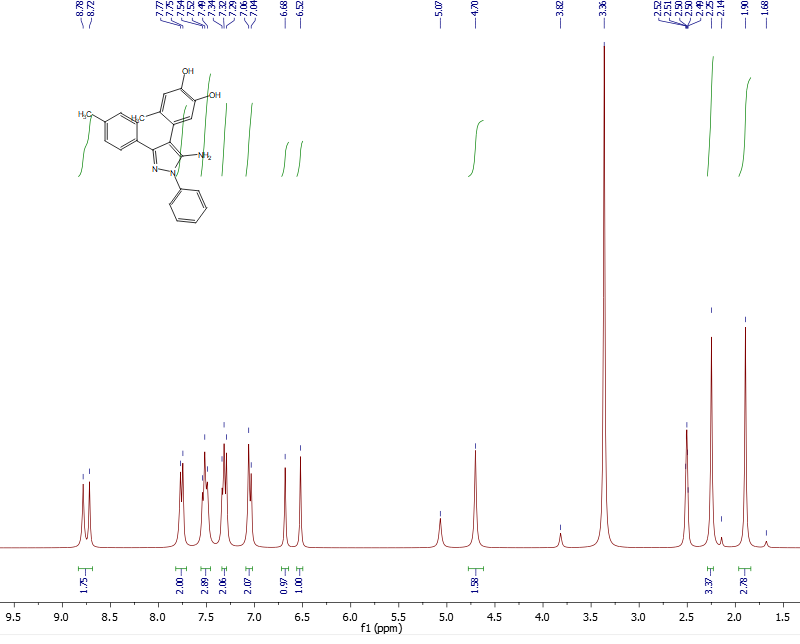


^1^HNMR (300 MHz, DMSO-d_6_) of compound **3e**


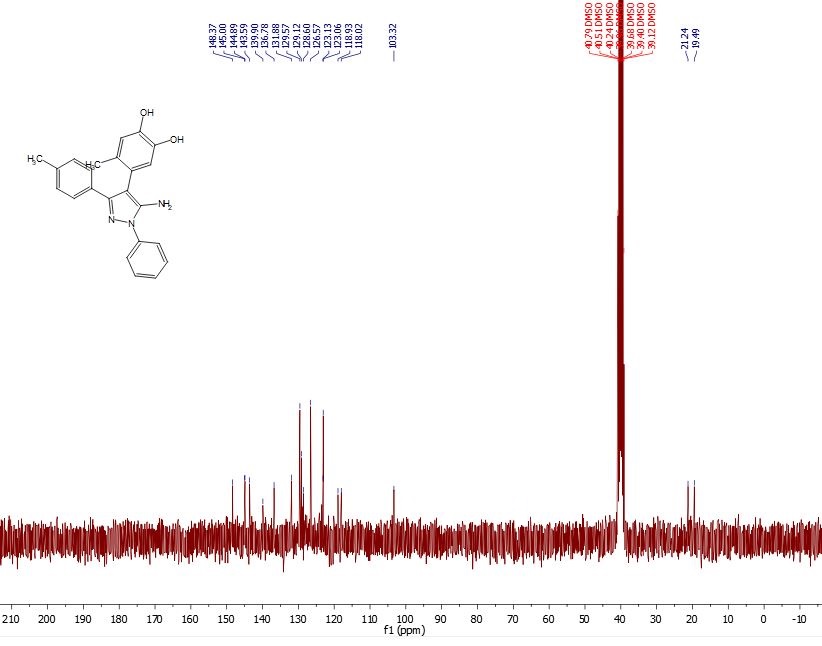


^13^CNMR (75 MHz, DMSO-d_6_) of compound **3e**

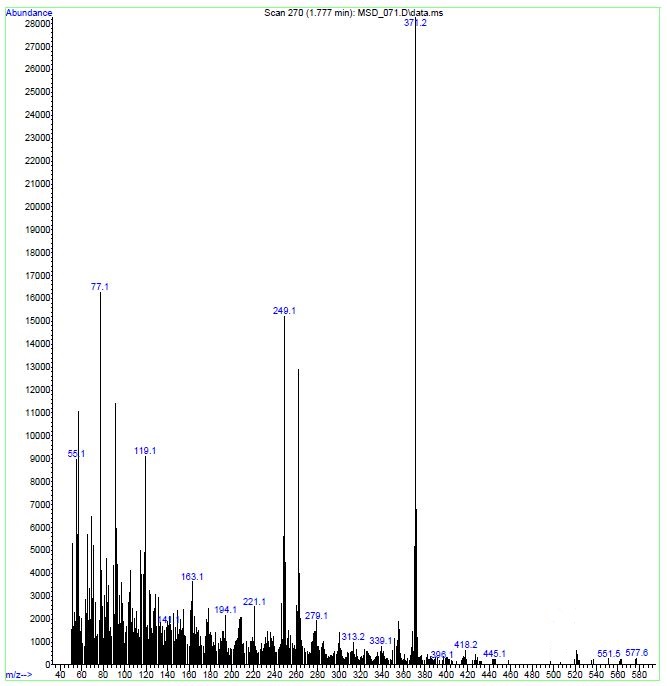


Mass spectra of **3e**


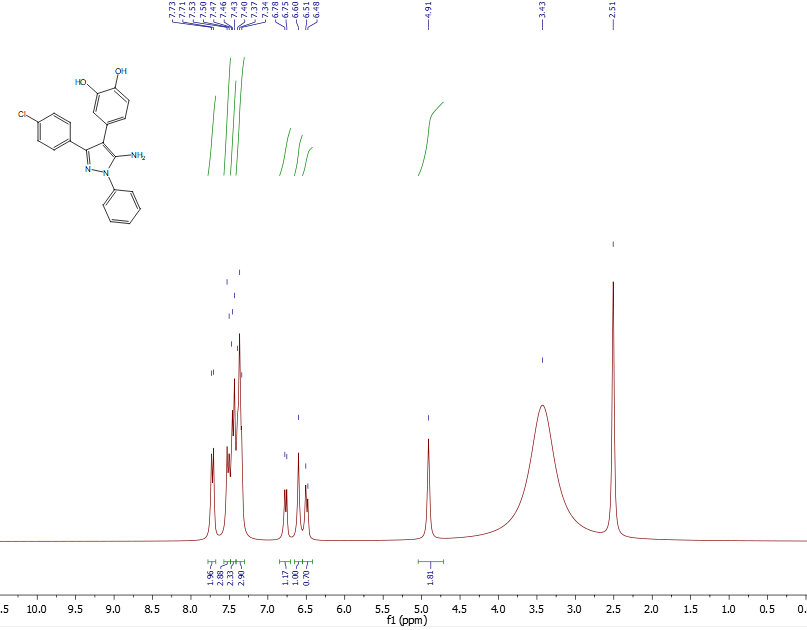


^1^HNMR (300 MHz, DMSO-d_6_) of compound **3f**


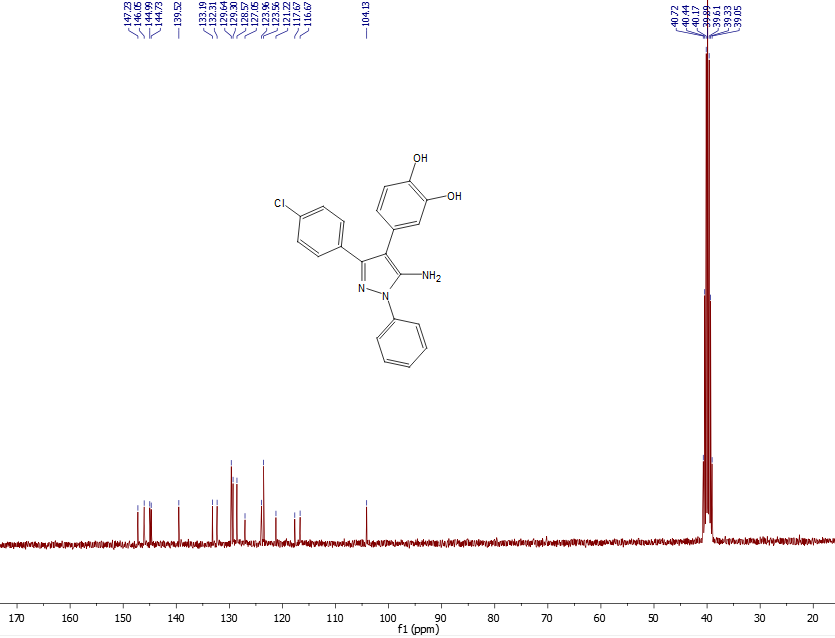


^13^CNMR (75 MHz, DMSO-d_6_) of compound **3f**

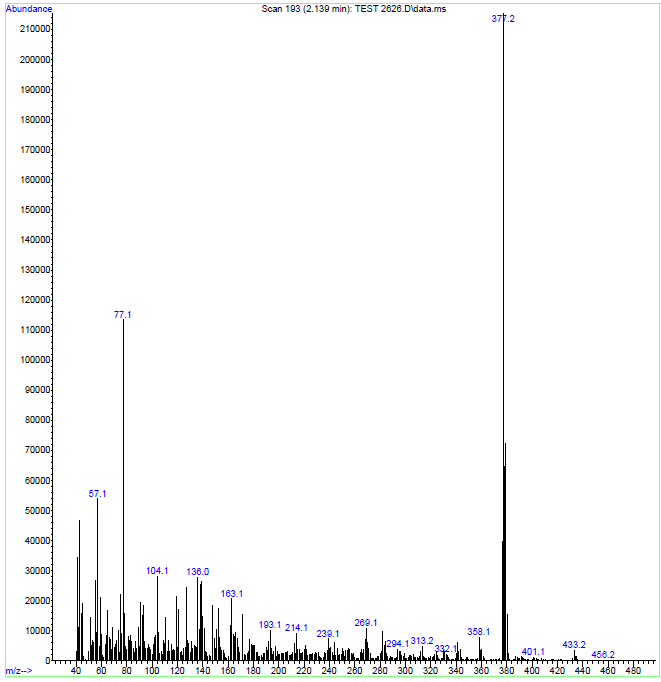


Mass spectra of **3f**


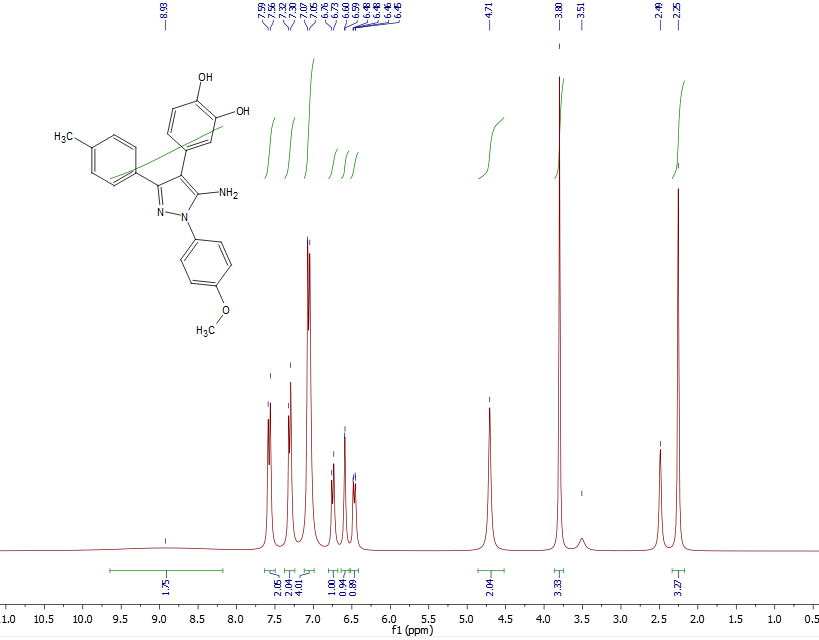


^1^HNMR (300 MHz, DMSO-d_6_) of compound **3g**


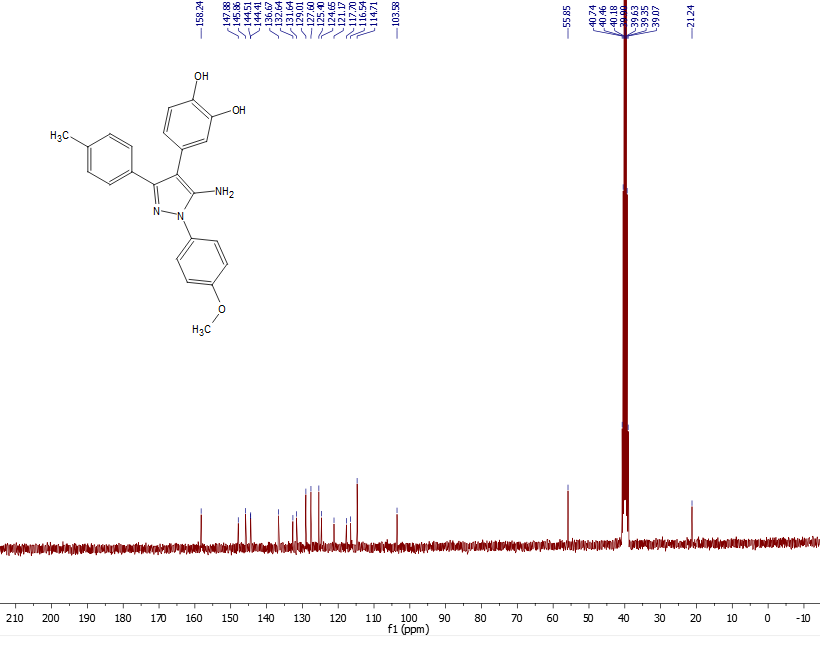
^13^CNMR (75 MHz, DMSO-d_6_) of compound **3g**

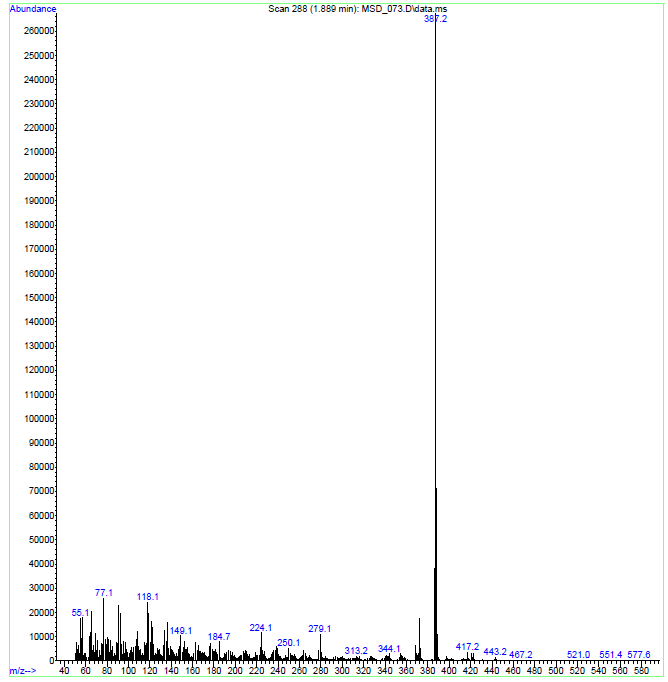


Mass spectra of **3g**


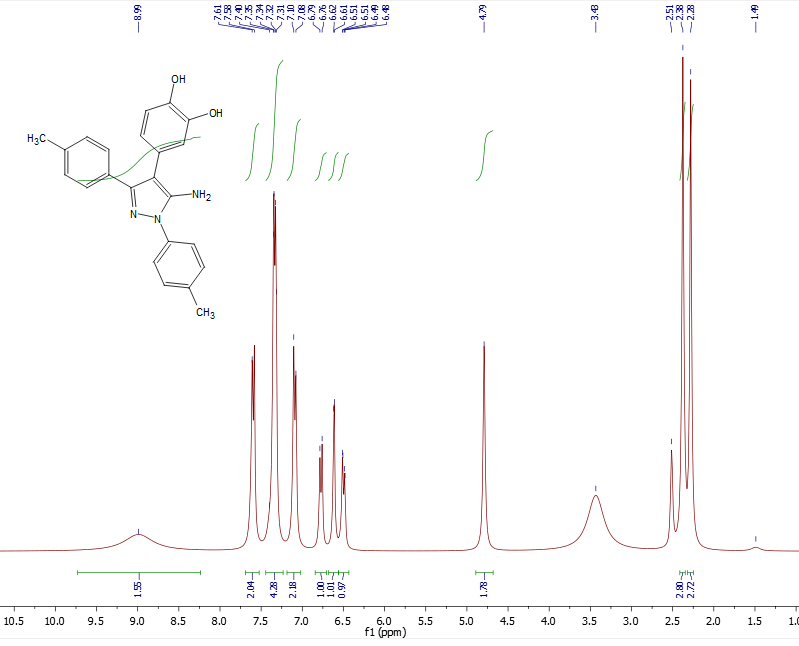
^1^HNMR (300 MHz, DMSO-d_6_) of compound **3h**


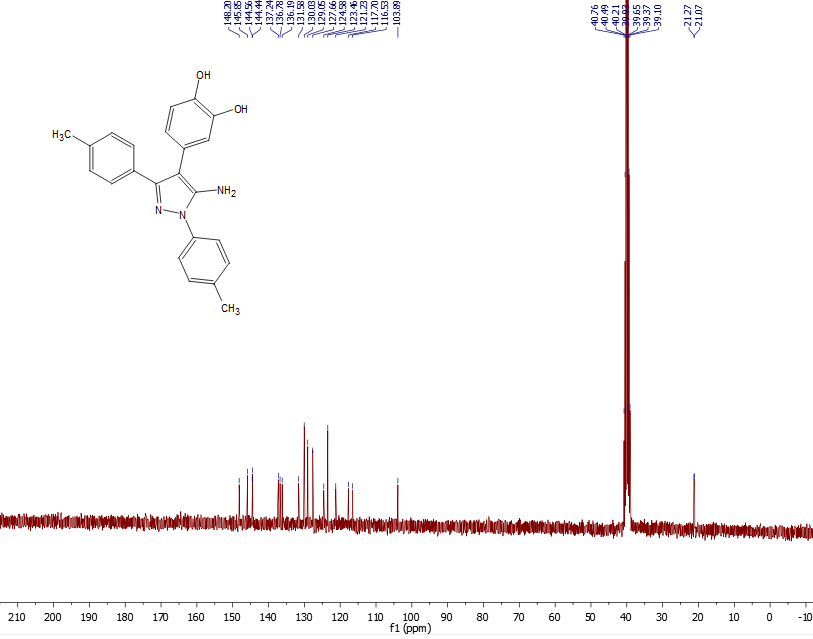
^13^CNMR (75 MHz, DMSO-d_6_) of compound **3h**

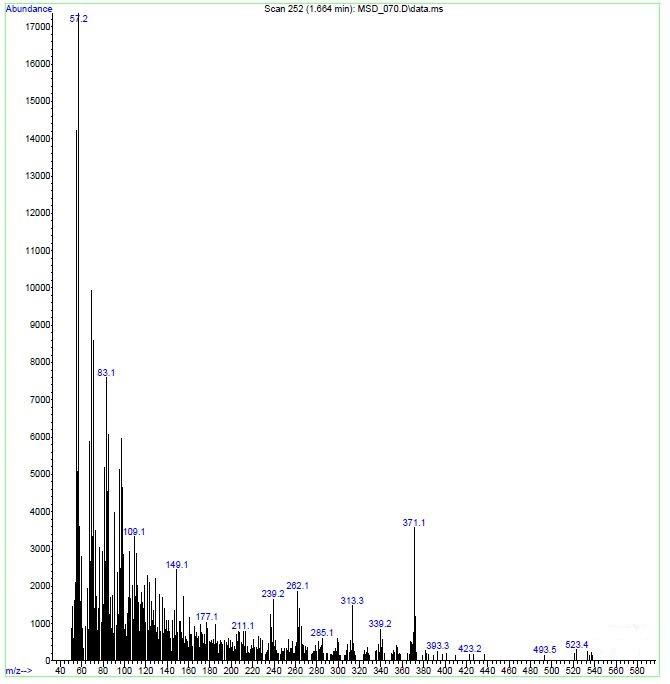


Mass spectra of **3h**


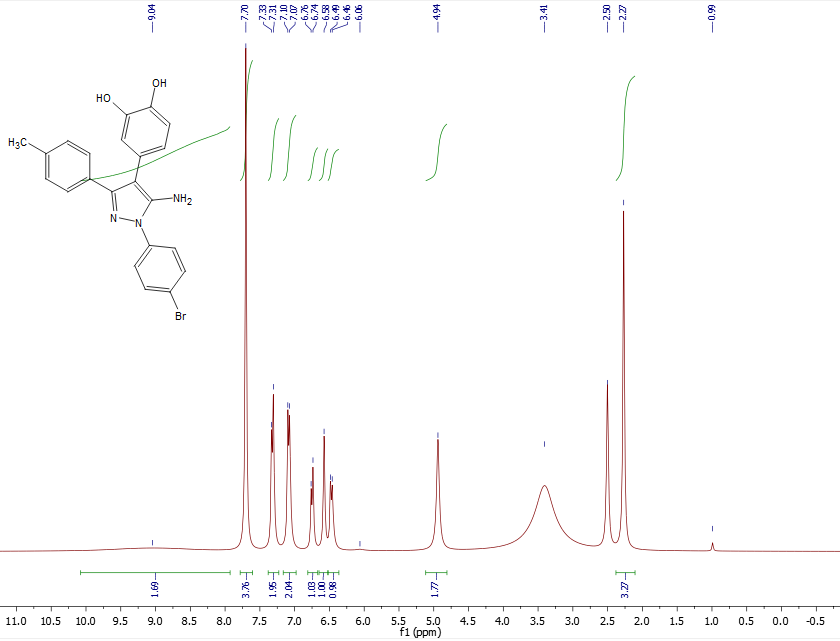


^1^HNMR (300 MHz, DMSO-d_6_) of compound **3i**


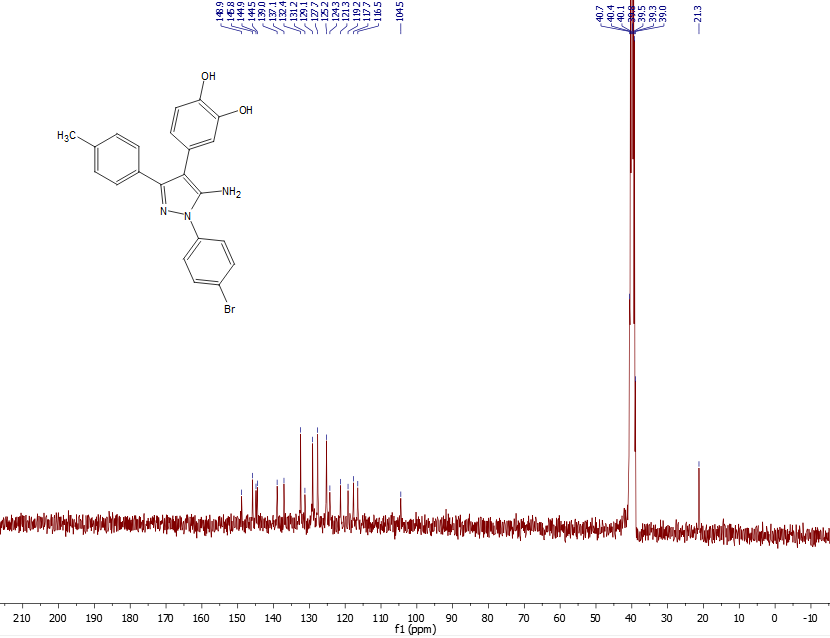


^13^CNMR (75 MHz, DMSO-d_6_) of compound **3i**

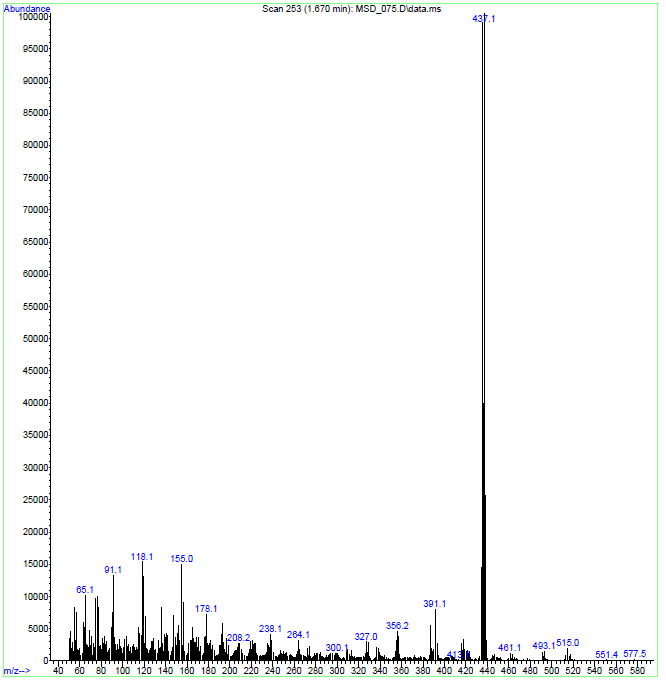


Mass spectra of **3i**


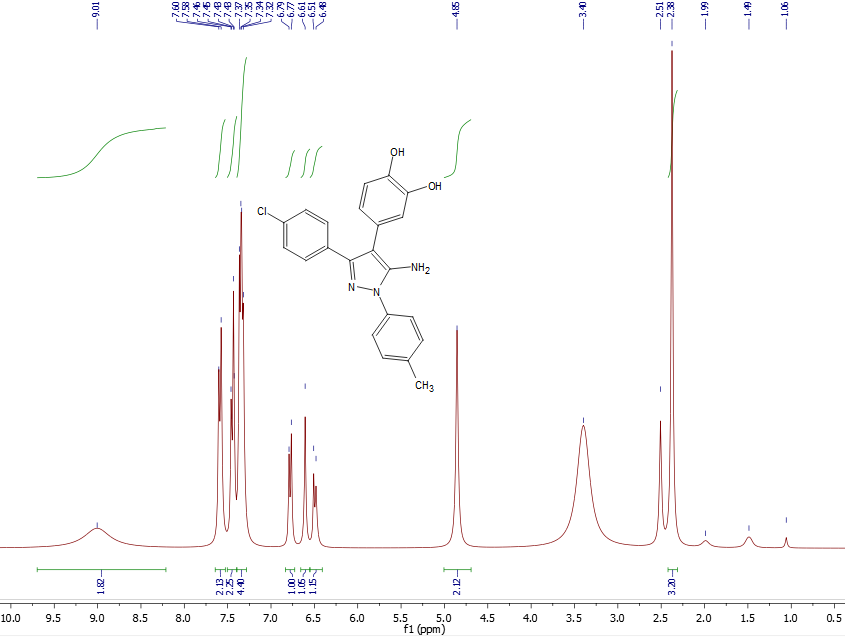
^1^HNMR (300 MHz, DMSO-d_6_) of compound **3j**


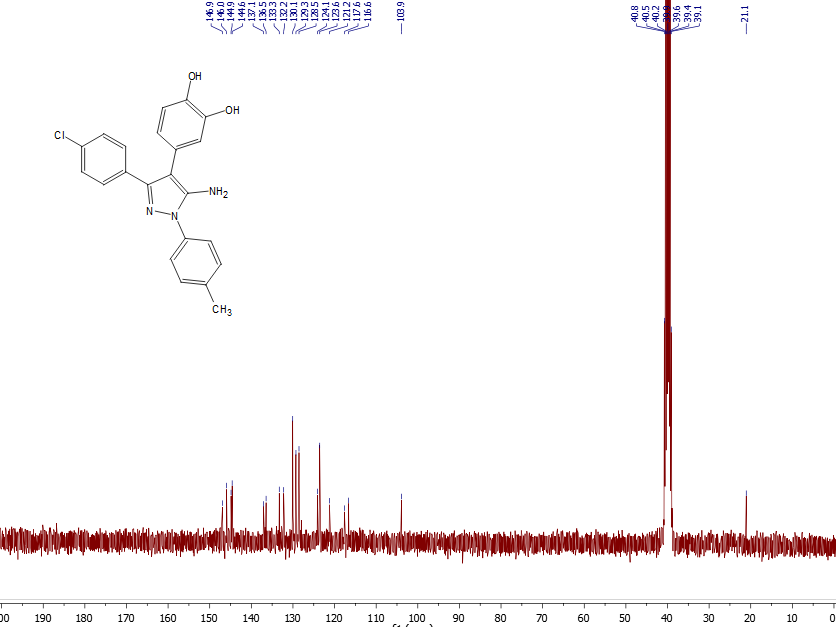
^13^CNMR (75 MHz, DMSO-d_6_) of compound **3j**

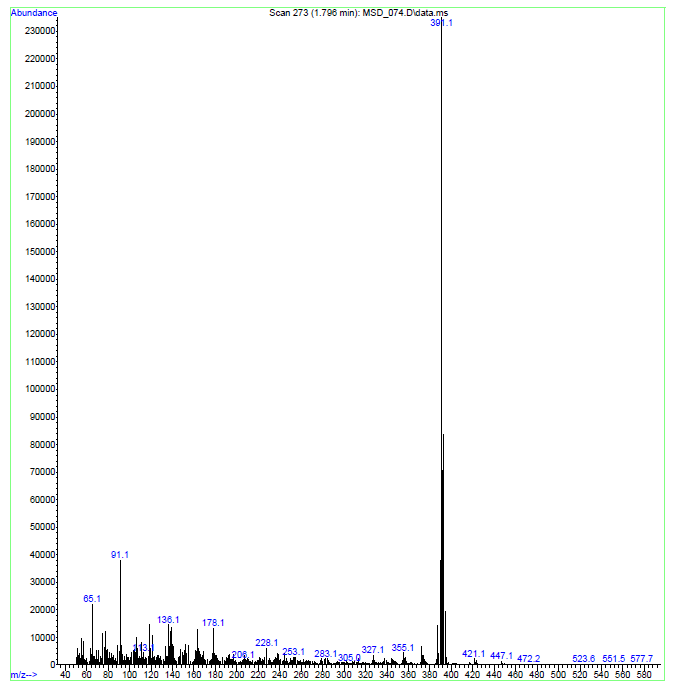


Mass spectra of **3j**
